# Tight Regulation of the *intS* Gene of the KplE1 Prophage: A New Paradigm for Integrase Gene Regulation

**DOI:** 10.1371/journal.pgen.1001149

**Published:** 2010-10-07

**Authors:** Gaël Panis, Yohann Duverger, Elise Courvoisier-Dezord, Stéphanie Champ, Emmanuel Talla, Mireille Ansaldi

**Affiliations:** 1 Laboratoire de Chimie Bactérienne, CNRS UPR9043, Institut de Microbiologie de la Méditerranée, Marseille, France; 2 Aix-Marseille Université, Marseille, France; The University of North Carolina at Chapel Hill, United States of America

## Abstract

Temperate phages have the ability to maintain their genome in their host, a process called lysogeny. For most, passive replication of the phage genome relies on integration into the host's chromosome and becoming a prophage. Prophages remain silent in the absence of stress and replicate passively within their host genome. However, when stressful conditions occur, a prophage excises itself and resumes the viral cycle. Integration and excision of phage genomes are mediated by regulated site-specific recombination catalyzed by tyrosine and serine recombinases. In the KplE1 prophage, site-specific recombination is mediated by the IntS integrase and the TorI recombination directionality factor (RDF). We previously described a sub-family of temperate phages that is characterized by an unusual organization of the recombination module. Consequently, the *att*L recombination region overlaps with the integrase promoter, and the integrase and RDF genes do not share a common activated promoter upon lytic induction as in the lambda prophage. In this study, we show that the *intS* gene is tightly regulated by its own product as well as by the TorI RDF protein. *In silico* analysis revealed that overlap of the *att*L region with the integrase promoter is widely encountered in prophages present in prokaryotic genomes, suggesting a general occurrence of negatively autoregulated integrase genes. The prediction that these integrase genes are negatively autoregulated was biologically assessed by studying the regulation of several integrase genes from two different *Escherichia coli* strains. Our results suggest that the majority of tRNA-associated integrase genes in prokaryotic genomes could be autoregulated and that this might be correlated with the recombination efficiency as in KplE1. The consequences of this unprecedented regulation for excisive recombination are discussed.

## Author Summary

Temperate bacteriophages are widespread bacterial viruses that have the ability to replicate passively in their hosts as long as no stressful conditions are encountered, a process called lysogeny. Prophage-encoded genes may benefit the host in several ways such as providing resistance to antibiotics, increased pathogenicity, or increased fitness. Most temperate phages insert their genome into the host's chromosome by site-specific recombination. After prophage induction, usually under stressful conditions, the excisive recombination constitutes a key step toward productive phage development. In this paper, we study the regulation of integrase genes that encode the enzyme required for integrative as well as excisive recombination. We noticed that for prophages inserted in or near tRNA genes the orientation of the integrase gene relative to the tRNA is crucial for its regulation.

## Introduction

Temperate bacteriophages are characterized by their ability to maintain their genome into the host, a process called lysogeny. Most temperate phages integrate their genome into the host's chromosome, becoming prophages. Alternatively, circularized phage genomes are maintained as episomes. Once integrated, the now so-called prophage is stable and replicates passively with its host genome. This situation can continue as long as outside conditions do not become threatening for the host, and therefore for the virus. Prophages are indeed able to detect many stressful signals, such as DNA damage, excessive heat or pressure [1]–[3]. By “listening” and hijacking the host's response to various stresses, prophages behave like perfect stress biosensors. Once the prophage is induced, the process of lysogeny escape is engaged, and the phage enters a lytic mode of development [1]. A crucial event in this process is the excision of the prophage from the host's chromosome. Replication of the viral genome follows, as well as the synthesis and the assembly of the virion proteins. Thus, excisive recombination is a highly regulated process that relies on two different levels of regulation: (i) protein activity, through the control of directionality by a recombination directionality factor (RDF), and (ii) protein synthesis via the coordinated expression of the integrase and RDF genes.

**Figure 1:**
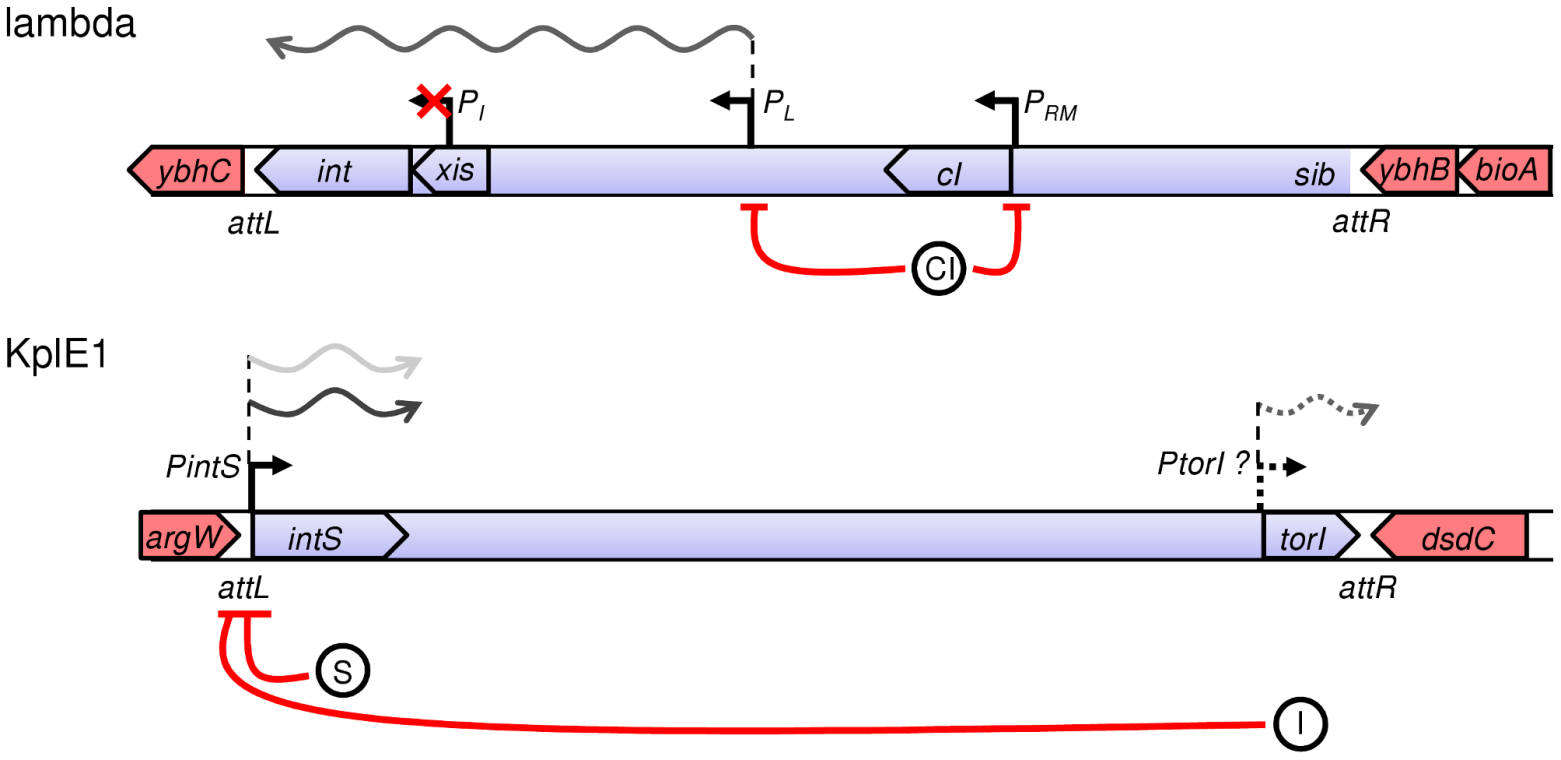
Recombination modules and regulatory features of integrase genes in lambda and KplE1 prophages. Regulatory features of the lambda integrase were adapted from [1]. Promoters of interest are indicated with black arrows; in the case of the non-characterized P*torI* promoter, the arrow is a dotted line. The *E. coli* chromosome is in red, whereas the prophages' genomes are in blue. The orientation of the genes is indicated with large arrows. Repression is represented by red lines, and the proteins involved in repression are indicated by circles (I, TorI; S, IntS; CI). Transcription patterns for the integrated state (light gray) and for the excising state (dark gray) are illustrated.

Temperate bacteriophages use site-specific recombination to integrate into and excise their genomes out of the host genomes. Integration consists of a strand exchange between the recombination region *att*P on the phage genome and *att*B on the bacterial chromosome leading to the formation of the recombined halves *att*L and *att*R at the junctions between the bacterial chromosome and the integrated phage genome (Figure 1). Lambda phage integrase has been extensively studied for its role in site-specific recombination and is essential for lysogeny establishment as well as for the transition to productive lytic development (reviewed in [4], [5]). The Int tyrosine recombinase catalyzes integrative and excisive recombination [6], [7]. Xis acts as a recombination directionality factor (RDF) as it bears no catalytic activity but rather directs the Int-driven reaction toward excision [8]. Xis plays an architectural role in the formation of the excisive intasome by binding and bending DNA, and prevents reintegration of the excised phage genome [9]–[11]. Precise stoechiometry of Int and Xis proteins is required for the correct assembly of the intasome nucleoprotein complex [12]. Since the organization of the protein binding sites of the *att* regions is not conserved, this suggests that the intasome architecture may vary according to the number and orientation of the recombination protein binding sites [13]. The phage-encoded integrase is a hetero-bivalent DNA binding protein in which the N- and C-terminal domains bind to different DNA substrates. The C-terminal domain, where the catalytic activity takes place, binds to and recombines the identical core-type sequences present in *att*P and *att*B, or in *att*L and *att*R, depending on the direction of the reaction considered [14]–[16]. The N-terminal domain binds to arm-type sequences [17], and this binding allows the assembly of the intasome, the nucleoprotein complex for site-specific recombination. Host-encoded proteins are also involved in this process, including IHF and Fis that bind and bend DNA in order to assist intasome formation [9], [18]–[20]. Recombination occurs through pair-wise exchange of four DNA strands between two *att* substrates. A four-way Holliday junction is formed upon the exchange of one pair of strands and then resolved after the DNA cleavage activity is switched from one pair of strands to another [21]–[24]. In all temperate phages, site-specific recombination events are believed to be identical; however, the organization of the *att* regions varies from one family of phages to another according to the number and orientation of the recombination protein binding sites. This suggests that the assembly and final composition of the intasome might follow different paths to eventually end with the same recombination reaction.

The KplE1 prophage (also named CPS-53) is a defective prophage integrated into the *argW* tRNA gene in *E. coli* K12 (Figure 1). The prophage's remaining genome (10.2 kb) contains 16 open reading frames (ORF) bordered by a duplicated core sequence of 16 nucleotides (CTGCAGGGGACACCAT). None of these ORFs seems to encode a repressor consistent with the finding that KplE1 is not SOS-inducible (M. Ansaldi, unpublished observation). Despite the small remnant genome, the KplE1 prophage can be excised *in vivo* [13], [25]. The KplE1 recombination module has been analyzed, and indeed it contains all the elements required for site-specific recombination to occur, including RDF and integrase genes as well as the *att*L and *att*R recombination regions [26]. This recombination module is highly conserved in several enterobacteria phage genomes such as CUS-3 and HK620 that infect *E. coli* strains K1 RS218 and TD2158, respectively, and Sf6, which infects *Shigella flexneri*, as well as in prophages present in *E. coli* strains APEC-O1 and UTI89 [27]–[32]. One advantage of studying the KplE1 prophage is that we can dissect the excisive recombination and its regulation *in vivo* independently of prophage induction since the CI regulator module is missing in KplE1. Directionality of the site-specific recombination has been studied using KplE1 DNA substrates as well as HK620 substrates and requires the RDF protein TorI to direct the recombination reaction towards excision [26]. One prominent feature of the KplE1 recombination is the orientation of the *intS* gene relative to the *att*L region (Figure 1). Indeed, the *intS* gene is transcribed from a dedicated promoter that overlaps with the *att*L region. In λ, *int* gene expression depends on the activity of two promoters *P_I_* and *P_L_* [1], [5]. While lysogeny is established, *int* expression relies on the *P_I_* promoter located in the *xis* gene and allows transcription of *int* independently of *xis*. Therefore, this promoter is used to establish lysogeny and ensures that more Int than Xis is being made [33]. During the escape from lysogeny, *xis* and *int* are co-transcribed as a consequence of P_L_ promoter activation and N antitermination (Figure 1). The differential expression of Int by these two promoters depends upon a site (*sib*) located distal to the *int* gene. Thus, lower amounts of Int are made, and Xis production is not affected by this element [34].

Based on the localization and orientation of the *intS* promoter that overlaps the *att*L recombination region (Figure 1), we performed preliminary experiments that led us to conclude that the *intS* gene is negatively autoregulated and poorly expressed during the exponential growth phase [26]. In this study, we further investigate the regulation of the *intS* gene in relation to the recombination efficiency. We provide *in silico* evidence that a majority of integrase genes associated with tRNA inserted prophages are predicted to negatively autoregulate. This prediction was subsequently confirmed *in vivo* with several examples. As a consequence, the integrase gene appears constantly expressed at a low level in KplE1, and the control of excisive recombination seems to rely only on the RDF expression rather than on a coordinate expression of the integrase and RDF genes.

## Results

### Experimental determination of the *intS* transcription start site

**Figure 2:**
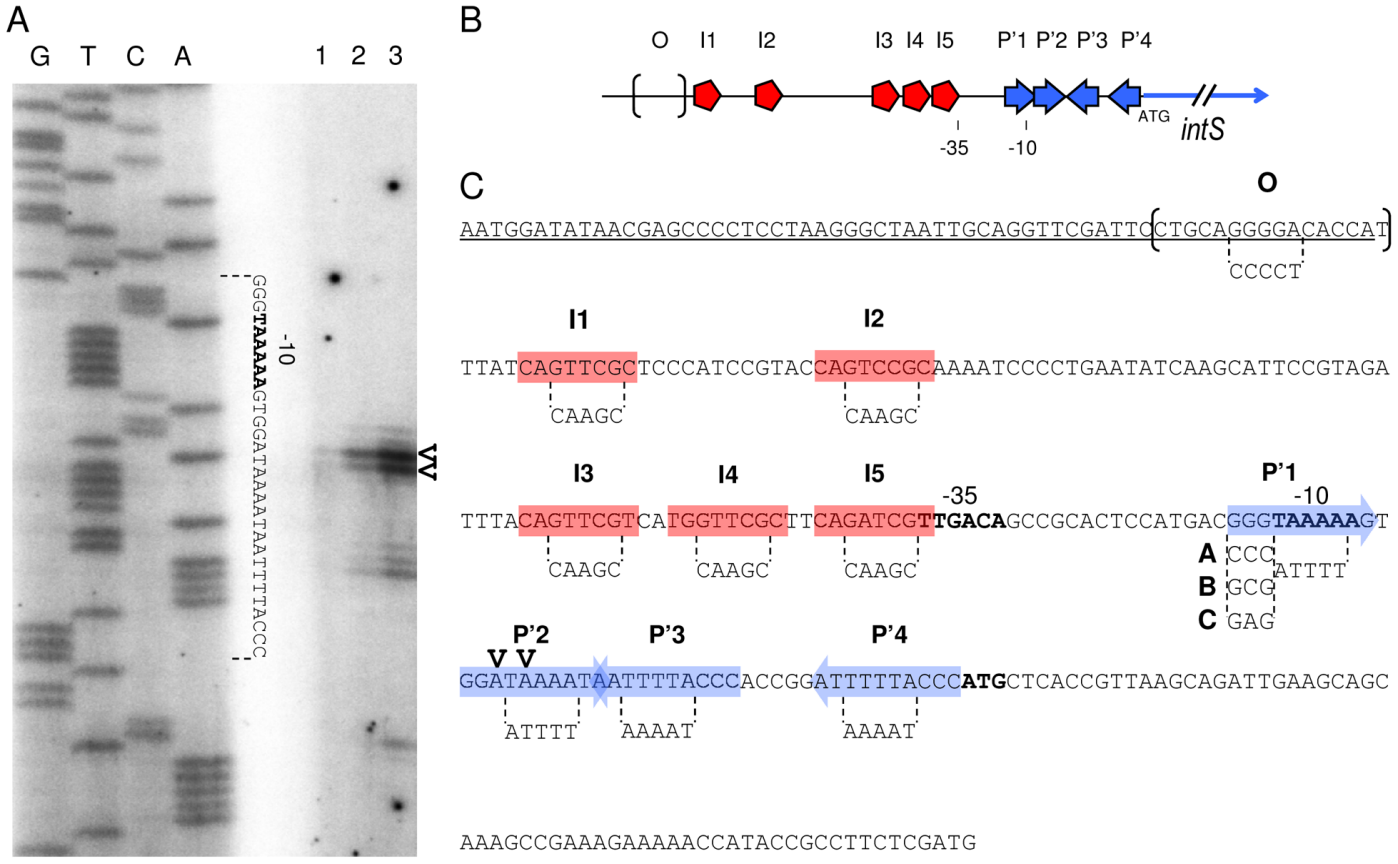
Primer extension analysis and mutagenesis strategy of the *intS* promoter. A. The labeled primer was annealed to RNA extracted from MC4100 (lane 1), LCB1024 (Δ*intS*, lane 2) and LCB1019 (ΔKplE1 prophage) harboring p*att*L-gfp plasmid (lane 3) strains grown aerobically and extended with reverse transcriptase. Lanes G, T, C and A are a sequencing ladder of the *att*L DNA region. A complementary sequence is indicated between *dotted lines*. B. Schematic representation of IntS and TorI binding sites on *attL* that overlap the *intS* promoter region (*I*, TorI; *P*', IntS arm-type and *O*, IntS core-type). The −35 and −10 boxes and ATG of *intS* are indicated. C. *intS* promoter sequence. This sequence corresponds to the *att*L region cloned as a reference for P*intS* promoter studies (p*att*L-gfp, positions −223 to +64 relative to the ATG). The *bold letters* show the putative −35 and −10 boxes and ATG (2464.565 kb on the MG1655 *E. coli* chromosome) of the *intS* gene. Protein binding sites' sequences are indicated (*red boxes*, TorI; *blue arrows*, IntS arm-type; and *brackets*, IntS core-type) as well as the two initiation transcription sites (V). The mutagenesis of the protein binding sites was performed by overlap extension PCR to generate mutations (*). Substitutions are indicated between *dotted lines*. Contrary to the *P*'1* mutation that affects the consensus of IntS arm-type and the −10 box, *P*'*1-*(*A*, *B* or *C*) constructions do not affect the −10 box. The 3′ end of the *argW* tRNA gene sequence is underlined (13 nucleotides of *argW* are missing at the 5′ end).

Previous work described the P*intS* promoter based on sequence analysis of the region upstream from the ATG starting codon [26]. This allowed the identification of putative −10 and −35 sequences close to the consensus sequences recognized by the σ70-RNA polymerase holoenzyme (TAaAAa and TTGACA, respectively) (Figure 2C). To show that the RNA polymerase actually utilizes this promoter to start *intS* transcription, we experimentally determined the *intS* transcription start site. Primer extension analysis was performed using total RNAs extracted from a wild-type as well as an *intS* strain, annealed with a labeled primer hybridizing downstream from the *intS* ATG (see Materials and Methods for details). In the presence of IntS (Figure 2A, lane 1), extension products were scarcely apparent. However, in the *intS* background (Figure 2A, lane 2) we observed two main extension products, indicating that transcription started at T and A residues at positions 2464536 and 2464537 on the *E. coli* chromosome, respectively. These transcription start sites are correctly located relative to the σ70-RNA polymerase holoenzyme binding sites, and the A at position 2464537 is perfectly positioned relative to the −10 box [35]. This latter transcription start site was also detected in a genome-scale analysis of transcription in *E. coli* [36]. Altogether, these experiments confirmed the previous localization of the *intS* promoter and the downregulation of the *intS* gene by its own product.

### Expression of the P*intS* promoter *in vivo*

The *intS* promoter, due to its location, obviously overlaps with the *attL* recombination region, and thus overlaps with IntS and TorI binding sites as previously characterized [26] (Figure 2C). In that study, we showed that the *intS* transcript originating at the chromosomal P*ints* promoter was five-fold more abundant in an *intS* background than in a wild-type strain. To study the influence of each protein binding site on P*intS* regulation *in vivo*, an accurate method was needed to quantify gene expression that would allow easy mutagenesis of the protein binding sites. We chose to use a *gfp* fusion-based vector (pUA66) that contains a sc101 replication origin, which leads to a low copy number (3 to 4 copies in the logarithmic growth phase) of the plasmid *in vivo* to avoid titration of the regulators [37]. The experiment was calibrated by cloning the entire *att*L region (positions 2464344 to 2464630 on the *E. coli* chromosome) in the pUA66 vector in order to measure p*att*L-*gfp* expression in various genetic backgrounds. Primer extension was used to control that transcription initiation occurred at the same site in this construct rather than in the chromosome (Figure 2A, lane 3). Indeed, the transcription start sites proved to be identical to those characterized on the chromosome when expressing the P*intS* promoter from a plasmid. Using this construct, we observed an increased transcription level of the P*intS* promoter compared to the chromosomal expression. This was likely due to a combination of two effects: the plasmid copy number and the fact that total RNAs were extracted from the LCB1019 strain that lacks the entire KplE1 prophage, and therefore the *intS* gene. Another explanation could be that this increase in transcription is linked to an increase in translation of the fusion. However, this is probably not the case because although integrase genes often contain rare codons that may slow down translation, a particular rare codon (AGA) is also present in the *gfp* gene.

**Figure 3:**
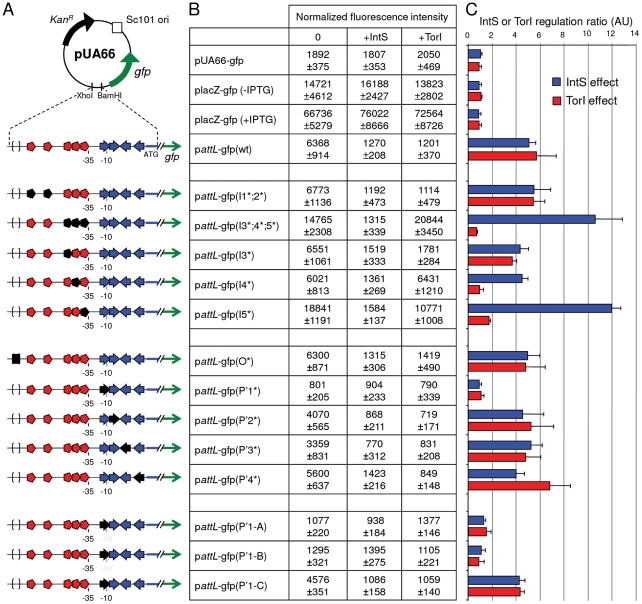
Regulation of the *intS* gene by both IntS and TorI proteins. A. Schematic representation of the *attL*-gfp transcriptional fusions. *Symbols* are as in Figure 2B and become black when IntS or TorI binding sites are mutated. B. LCB6007 (Δ*intS*)/pJF119EH (0), ENZ1734 (*wt*)/pJF119EH (+IntS) or LCB6007/pJFi (+TorI) strains were transformed with p*attL*-gfp(wt) and p*att*L-gfp (*) mutated plasmids. After overnight aerobic growth in the presence of 1 mM of IPTG for *torI* induction, the normalized promoter activity, emission at 521 nm/A_600_, was calculated (see Materials and Methods). C. IntS and TorI inhibition ratios onto the P*intS* promoter.

We measured the fusion expression with two different methods: direct fluorescence measurement (Figure 3), which gave a whole population measurement, and microscopic counting (Figure S1), which estimated the homogeneity of the fluorescent population. As indicated in Figure 3B, the *att*L*-gfp* wild-type fusion was expressed at a high level in the absence of IntS (6368±914 Units) and was repressed in the presence of IntS (1270±208 Units), leading to a repression ratio of ∼5 when the control ratio of *placZ-gfp* expression was close to 1 in the same conditions. This ratio of ∼5 is in complete agreement with the values we obtained by measuring *intS* expression from the chromosomal gene with quantitative RT-PCR [26], indicating that the fusion expression from several copies did not modify the regulatory ratio. Expression of the fusion was homogenous under all conditions, and the most resolved peaks were observed for cells producing TorI or IntS and therefore emitting little fluorescence (Figure S1). Thus, the results measured in the whole population (Figure 3) reflect homogenous expression of the fusion.

Looking at the recombination protein binding sites identified on *att*L (Figure 2C), it was obvious that some of the TorI RDF binding sites were also near the −35 sequence. We thus looked at a possible effect of TorI on *intS* expression. As indicated in Figure 3B, overexpression of TorI in an *intS* background led to a strong decrease in expression of the *patt*L*-gfp*(wt) fusion (compare 6368±914 with 1201±370 units). Taken together, these results show that *intS* expression is under the negative control of both TorI and IntS.

### Identification of the recombination protein binding sites involved in *intS* downregulation

The *att*L region contains five TorI binding sites organized in two blocks (Figure 2C, red symbols). The first one (I1;2), encompasses sites I1 and I2 that are separated by 12 nucleotides (positions 2464409 to 2464436). The second block is composed of three binding sites (I3;4;5) separated by 2 nucleotides (positions 2464472 to 2464499). We mutated each site by changing the sequences GTTCG, GATCG, GTCCG into CAAGC. When both sites of block I1;2 were mutated we did not observe any effect on the TorI mediated downregulation of *intS* (p*attL*-gfp(I1*;2*)) with a repression ratio of 5.4 (Figure 3). In contrast when the sites of the second block were changed, p*attL*-gfp(I3*;4*;5*), TorI was no longer able to repress the expression of the fusion, meaning that at least one of these sites was important for repression. We thus measured the effect of each site independently. If the mutation of site I3 had little effect on the repression ratio (4.7), the mutations of sites I4 and I5 led to expression of the fusion independent of the presence of TorI (repression ratios of 1.0 and 1.7, respectively). These two sites are the closest to the −35 sequence and are therefore appropriate candidates for mediating TorI repressor activity. We observed increased basal expression of the fusion (from 6,400 to 15,000–19,000 units) when the I5 site was mutated. This effect is probably due to the change in the nucleotides adjacent to the −35 sequence that results in a promoter-up phenotype.

We then studied the implication of the arm-type binding sites (*P*' sites, blue symbols in Figure 2B and 2C) of the integrase. For the *P*' sites the conserved motif TAAA present in all *P*' sites was changed into its complement ATTT. Interestingly, none of the individual mutations led to derepression of the fusion; indeed, in all cases (except for the *P*'1*, see below), the repression ratio ranged from 4.0 to 5.2 (Figure 3B and 3C). The *P*'*1* site's influence was more difficult to study since it overlapped with the −10 sequence (Figure 2C). Thus, any mutation of the conserved motif led to an inactive promoter whose measured fluorescence did not exceed that of the promoter-less fusion (Figure 3, compare pUA66-gfp with p*att*L-gfp(P'1*)). Additional constructs were made to avoid this effect on the promoter activity; however, any change we made that altered IntS binding also affected promoter activity (Figure 3, constructs p*att*L-gfp(P'1-A and B)), and in the case the latter was not affected (p*att*L-gfp(P'1-C)), neither was the down regulation of *intS*. In a control experiment, we mutated the core site, which is the binding site for the catalytic domain of the integrase, and this construct showed an unaltered repression phenotype (Figure 3) as well as IntS binding similar to that observed with the wild-type sequence (data not shown and [13]). Altogether, these data demonstrate that both TorI and IntS negatively regulates the *intS* gene *in vivo* and point to the TorI and IntS sites located near the −35 and −10 sequences as being responsible for the downregulation of *intS* gene expression. These results also show that the *intS* gene is tightly regulated and is thus expressed at a low level under all tested growth conditions.

### A critical IntS concentration is required for efficient excisive recombination

**Figure 4:**
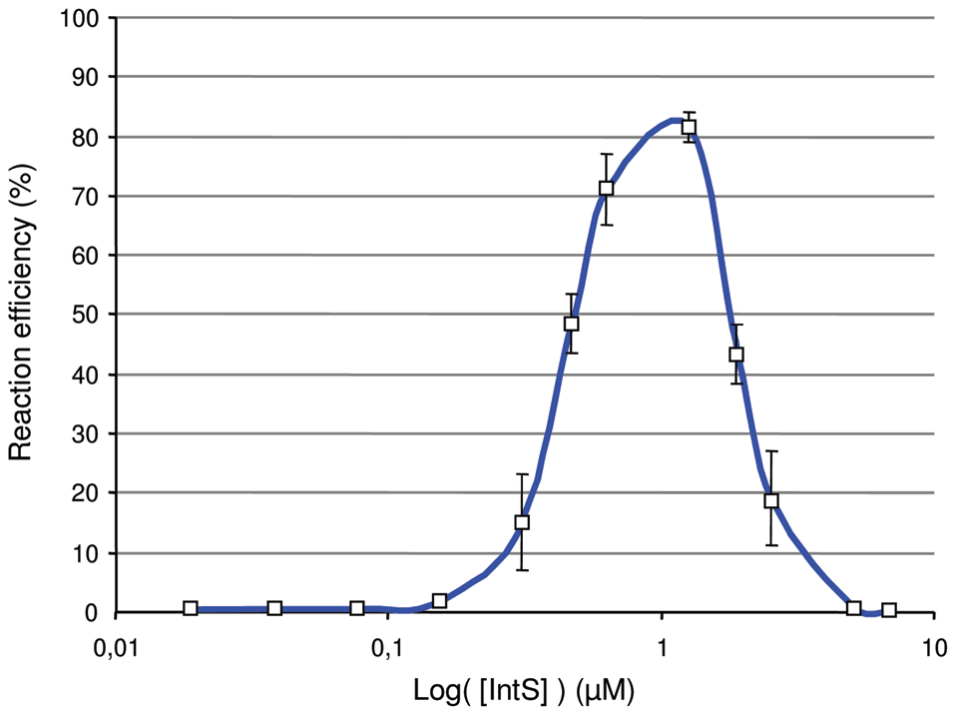
*In vitro* effect of IntS concentration in excisive recombination. *In vitro* excisive recombination was performed with *att*L and *att*R linear substrates at equimolar concentration (32 nM). Purified TorI (1.6 µM) and IHF (0.25 µM) were added. Increasing concentrations of IntS were added (0.02 to 6.7 µM) and samples were incubated for 1 h at 37°C. Integration recombination efficiency was determined by Q-PCR by using an *att*P standard curve. Measurements were carried out in triplicate and repeated at least three times.

One could ask about the “*raison d*'*être*” of this atypical integrase gene regulation compared with the lambda *int* gene. For that purpose, we measured the efficiency of the excisive recombination reaction *in vitro* as a function of the integrase concentration. Briefly, 32 nM of *att*L and *att*R linear substrates were incubated at 37°C for 1 h in the presence of constant concentrations of TorI and IHF (1.6 µM and 0.25 µM, respectively) and increasing concentrations of IntS (0.02 to 6.7 µM). The *att*P product was quantified by Q-PCR and the efficiency of the reaction was calculated as the percentage of substrates transformed into products. As the concentration of IntS increased, the efficiency of the reaction increased until a maximum level of ∼80% was achieved for an IntS concentration around 1 µM (Figure 4). However, when the IntS concentration exceeded 1.2 µM, we rapidly observed an inhibitory effect of IntS on the excisive reaction. Subsequently, the concentration range for which the efficiency of the reaction reached more than 50% was very narrow (0.8 µM up to 1.2 µM). These results show that to obtain the maximum efficiency in excisive recombination a precise integrase concentration is required.

**Figure 5:**
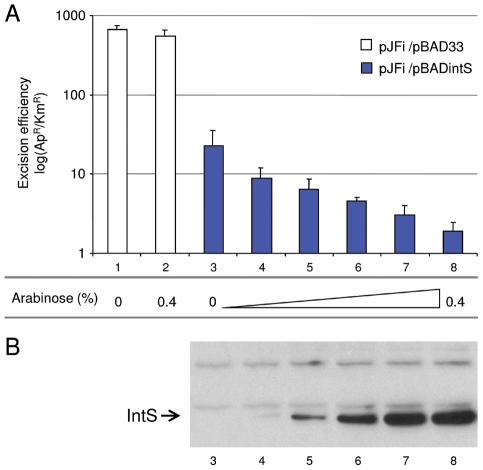
*In vivo* effect of IntS overexpression on the excision of KplE1 prophage DNA. A. Excision tests were performed in strain LCB6005 (*tfaS*::Km). pJFi and pBADintS encode wild-type TorI and IntS proteins, respectively. All *in vivo* excision experiments (see Materials and Methods) were performed in the presence of 1 mM IPTG for *torI* gene expression, and different amounts of arabinose were used for *intS* gene expression: 0% (lane 1 and 3), 0.00064% (lane 4), 0.0032% (lane 5), 0.016% (lane 6), 0.08% (lane 7) and 0.4% (lane 2 and 8). Excision efficiency is expressed as the ratio of ampicillin-resistant/kanamycin-resistant colonies (Ap^R^/Km^R^). B. IntS relative amount (lanes 3–8 in reference to A.) in crude extracts was analyzed after separation on a 12% SDS-PAGE with Western blot using polyclonal IntS antiserum. The IntS position is indicated with an arrow.

The effect of IntS overloading was then analyzed *in vivo*. Strain LCB6005 contains a Km resistance cassette in the tail fiber encoding gene (*tfaS*) of the KplE1 prophage, thus allowing an *in vivo* excision assay to be performed without any effect on the site-specific recombination process. This strain was transformed with the pJFi plasmid that contains the *torI* gene under the control of an IPTG inducible promoter as well as with the pBAD33 vector containing or not the *intS* gene under the control of an arabinose inducible promoter (pBADintS). Colonies were counted after the different cultures induced with IPTG were plated on LB medium containing ampicillin or kanamycin (see the Material and Methods section). Ap^R^ colonies are representative of the total number of cells since all contain the pJFi plasmid (Ap^R^), whereas Km^R^ colonies originate from cells that have kept the *tfaS::kan* marker, and thus the KplE1 prophage. As shown before [25], expressing *torI* from a multicopy plasmid (pJFi) is sufficient to promote excisive recombination. Indeed, in the strain containing the low copy vector alone (pBAD33), the maximal level of excision was achieved in the presence of TorI as revealed by a high Ap^R^/Km^R^ ratio (Figure 5A), and the addition of the arabinose inducer did not impede the reaction's efficiency. However, in the presence of the pBintS plasmid, even without adding the arabinose inducer, we observed dramatically decreased recombination activity (Figure 5A, compare bars 1 and 3). It is striking that, even at a concentration of integrase that could not be detected on a Western blot (Figure 5B, lane 3), i.e., in the absence of an inducer, the efficiency of the reaction underwent a 50-fold decrease. We explain this effect by the leakage of the pBAD promoter in the absence of glucose. Indeed, this promoter is induced in the presence of arabinose and repressed in the presence of glucose [38]. Since we do not use glucose in the medium, the pBAD promoter is not repressed, and some integrase is being made, although not sufficiently to immunodetect it. We therefore consider the empty vector as the actual negative control. Adding arabinose to the medium, which led to overproduction of IntS (Figure 5B, lanes 4 to 8), amplified this negative effect on the *in vivo* excision reaction. As a result, the *in vivo* recombination efficiency was negatively correlated with the increasing integrase concentration, thus confirming the results we obtained *in vitro*.

### Occurrence of predicted self-regulated integrase genes in prokaryotic genomes

**Figure 6:**
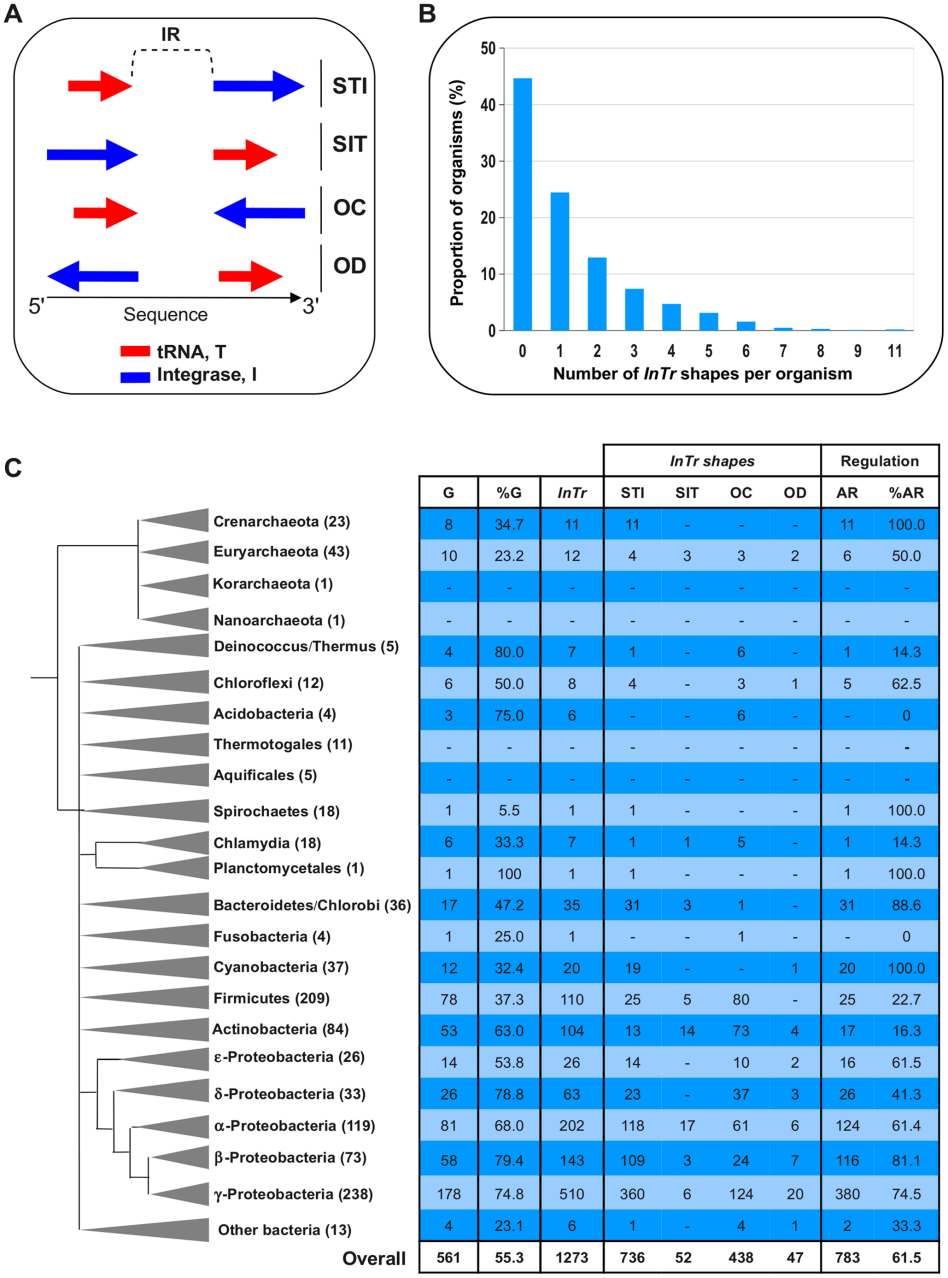
Abundance and distribution of *InTr* shapes. A. Types of *InTr* shapes. Arrows show the orientation of I (Integrase) or T (tRNA) with respect to the sequence orientation. STI, Same orientation and T precedes I; SIT, Same orientation and I precedes T; OC, Opposite orientation and Convergent; OD, Opposite orientation and Divergent. SelC, is the selenocysteinyl-tRNA gene. The intergenic region (IR) between I and T is indicated. B. Distribution of the *InTr* copy-number within prokaryotic genomes. C. Distribution of the *InTr* shapes within prokaryotic taxonomic groups. For each archaeal and bacterial main phyla, the numbers within parentheses indicate the number of complete genome organisms available for this study. G and % G, the number of genomes harboring at least one *InTr* shape, and the proportion (in percentage) compared to the overall genome of the phylum, respectively. *InTr*, the total number of *InTr* in the phylum and the different shapes types. AR, the overall number of *InTr* predicted to be autoregulated and the proportion (% AR) in percentage.

To address the general relevance of the negative autoregulation of the *intS* gene, a large-scale *in silico* analysis of tRNA-associated integrase genes was performed on the complete prokaryotic genomes available at that time. The *in silico* outline is described in the “Materials and Methods” section. Experimentally well-characterized integrases such as ^λ^Int and ^KplE1^IntS contain at least one of the three functional domains, Phage_integrase, Phage_integ_N, and Phage_integr_N, referred to as PF00589, PF09003 and PF02899 in the Pfam database, respectively. By using these functional domains as queries, we detected 8368 protein homologs within 1014 complete prokaryotic genomes, and 1273 of the corresponding integrase genes (15% of the total) are adjacent to a tRNA gene. These couples of tRNA-integrase genes (called *InTr* shape) constitute the primary data set used in this study (Table S1). *InTr* shapes were classified according to their gene coding orientation, leading to four different types of *InTr* shapes (Figure 6A): STI (Same orientation and T precedes I), SIT (Same orientation and I precedes T), OC (Opposite and Convergent orientation) and OD (Opposite and Divergent orientation). We then analyzed the distribution of the *InTr* copy-number per organism (Figure 6B) as well as the distribution of *InTr* shapes over the prokaryotic phylum (Figure 6C). A detailed analysis of these data is available in the Text S1. Overall analysis shows that the majority of the *InTr* shapes exhibits STI and OC shapes with 736 and 438 representatives, respectively. The other two classes (SIT and OD) occur relatively rarely (less than 8% in total) in the analyzed genomes. Therefore, the high occurrence of STI and OC shapes within the prokaryotes may highlight the functional importance of these shapes in microbial organisms.

### Relationship between the *InTr* shapes and integrase regulation

To study a possible correlation between the prevalence of *InTr* shapes and the autoregulation of the integrase genes as demonstrated for the *intS* gene, the number of putative autoregulated integrase genes was determined. Based on our experimental model, we proposed that STI and OD shapes should be subjected to autoregulation, since in these cases the integrase gene promoter overlaps with the recombination region, whereas SIT and OC shapes should show integrase gene expression independent of the integrase protein. Our *in silico* results indicated that *InTr* shapes containing Asn, Cys, Gln, Gly, Leu, Phe, SelC, and Ser tRNA genes were mainly predicted to autoregulated (Table S2). In contrast, the opposite conclusion can be drawn for *InTr* shapes containing Ile, Lys and Tyr tRNA genes, which is consistent with the observation that prophages are preferentially inserted in poorly expressed tRNA genes, probably to avoid a deleterious effect on cell fitness ([39]–[41]. A detailed analysis of the distribution of *InTr* shapes with respect to tRNAs in prokaryotic genomes is available in Figure S2 and Table S3. Out of the 1273 *InTr* shapes analyzed, 61.5% were detected as potentially autoregulated, most encoded within the Proteobacteria, Cyanobacteria, Bacteroidetes and Crenarcheota genomes (Figure 6C and Table S2). Thus, a situation that has rarely been described and studied in the literature is actually predominant in the sequenced prokaryotic genomes.

**Figure 7:**
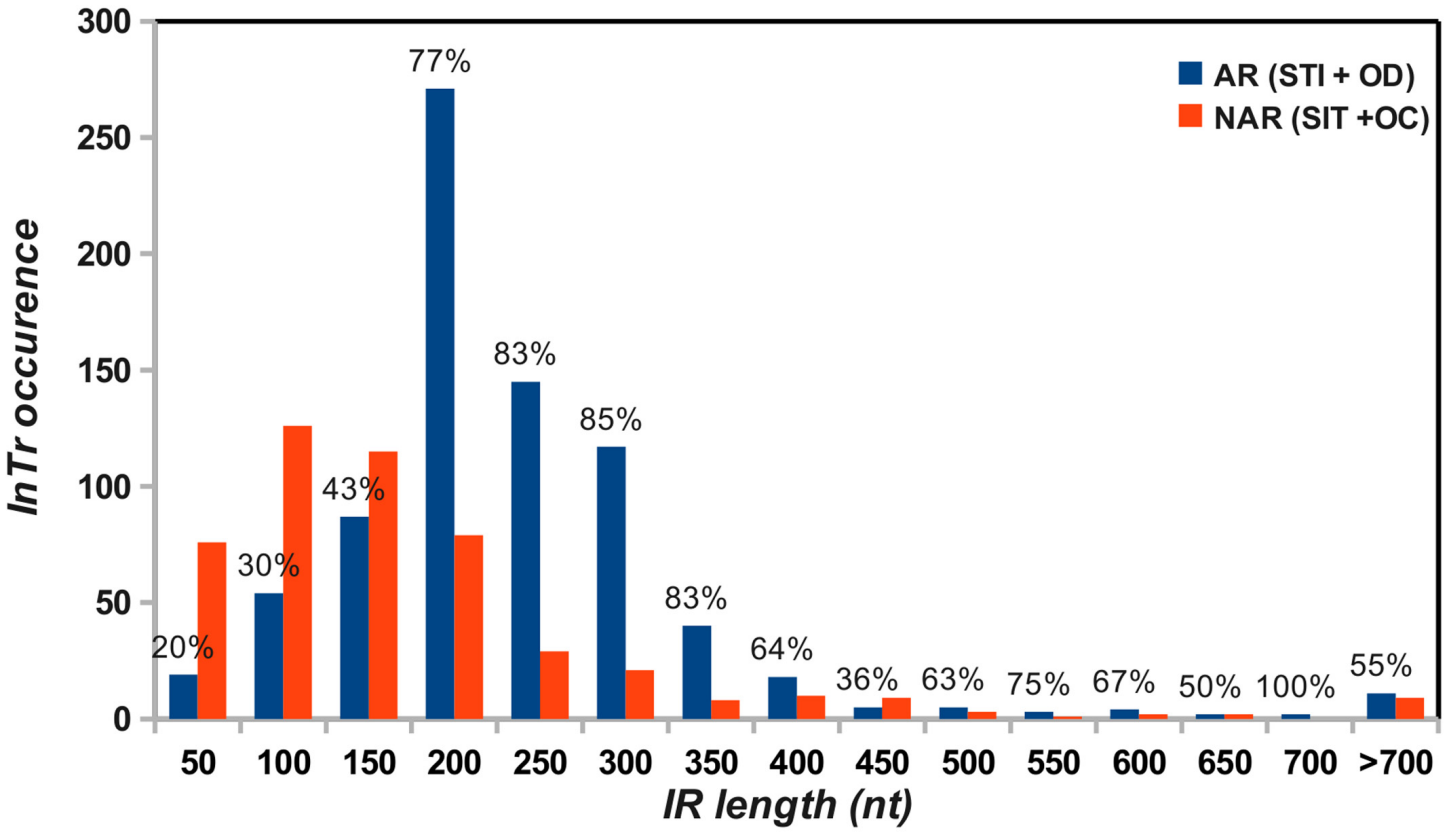
Distribution of the intergenic region lengths of putative autoregulated (AR) and non-autoregulated (NAR) *InTr* shapes. For each length interval, the proportion of the predicted autoregulated and non-autoregulated *InTr* shape (in percentage) is indicated.

We next addressed whether a relationship exists between the length of the intergenic region (IR, Figure 6A) and the fact that an integrase gene is predicted to be autoregulated. Therefore, the IR length was determined for each *InTr* shape, and the distribution of the obtained values was analyzed as a function of autoregulated and non-autoregulated *InTr* shapes (Figure 7). The lower values of the IR lengths are statistically associated with predicted non-autoregulated *InTr* shapes as the 95% confidence intervals of the mean IR length values are [157.5–158.4] for non-autoregulated *InTr* and [208.3–227.9] for predicted autoregulated *InTr*. These results clearly indicate that autoregulated *InTr* shapes are linked to large IRs. Our prediction is that autoregulation of the integrase mostly correlates with STI and OD shapes, and therefore the IR should be large enough to contain an entire *att*L region. As mentioned above, the average distance observed for predicted autoregulated *InTr* shapes [208.3–227.9] is perfectly compatible with the presence of an average *att*L region of 80–170 nucleotides.

Biological validation of the autoregulation of integrase genes involved in STI and OD *InTr* shapes.

**Figure 8:**
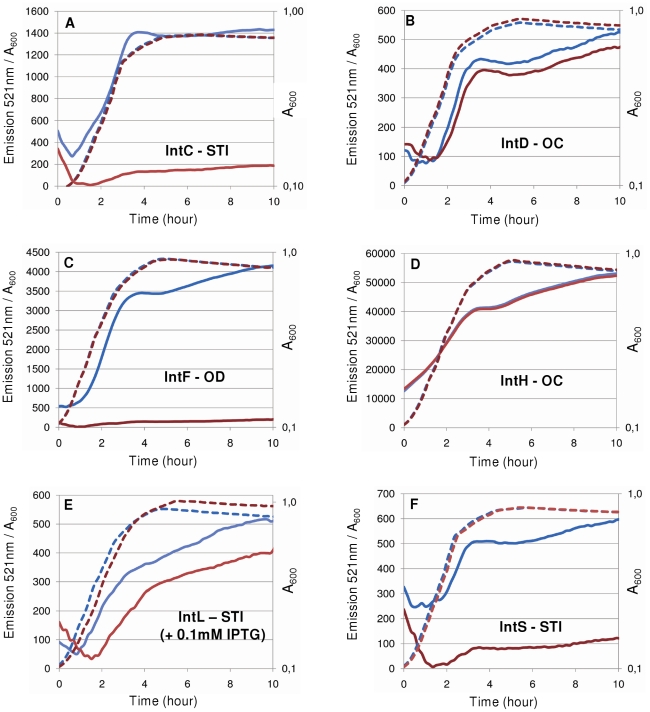
Regulatory patterns of various *E. coli* integrase promoters. GFP measurements were performed over time and normalized fluorescence intensities (emission at 521 nm/A_600_) are mentioned on the Y-axis (plain lines). Dotted lines represent the A_600_ values (X-axis, log(A_600_)). pJF119EH (blue lines) and pJF119EH containing the integrase genes (red lines) were co-transformed with the pUA66 plasmid containing the corresponding integrase promoter in the LCB1024 (Δ*intS*) strain for IntC (A) and IntS (F), the LCB6037 (Δ*intD*) strain for IntD (B), the LCB6038 (Δ*intF*) strain for IntF (C) and the ENZ1734 (*wt*) strain for IntH (D) and IntL (E) (see plasmids in Table 1 and details in the Materials and Methods section). IPTG (0.1 mM) was added to the culture to promote IntL production.

To validate the *in silico* predictions, we chose to study the expression of several integrase promoters from *E. coli* strains K12 MG1655 and O157:H7 EDL933. The promoters of the integrase genes were cloned into the pUA66 vector upstream of the *gfp* gene and the cognate integrase coding sequences were cloned into the pJF119EH vector (see plasmid list in Table 1). Regarding the *InTr* shapes, in addition to the well-characterized STI *argW-intS*, we studied 2 STI shape *argW*-*intC* (the *argW*-*intS* homologous shape in EDL933) and *selC-intL*, 2 OC shapes *argU-intD* (MG1655) and *thrW-intH* (EDL933) and 1 OD *ptwF-intF* (MG1655). Of these integrase genes, 3 are predicted to be autoregulated (*intC*, *intF*, and *intL*), and 2 should not exhibit autoregulation (*intD* and *intH*). To avoid the influence of the chromosomal copies of MG1655 integrase genes, we transformed both kinds of plasmids (the empty vector and the integrase encoding vector) in the appropriate deletion mutant, and when applicable, we used the MG1655 mutant for the EDL933 equivalent. As indicated in Figure 8, none of the OC shape associated integrase genes showed self-regulation, and the STI and OD shape integrase genes were negatively autoregulated. However, different regulation ratios were observed depending on the integrase gene considered. Interestingly, the pIntS-gfp fusion was repressed almost 15 times during the exponential growth phase (time point ∼2 h) whereas a repression ratio of 6 was measured during the stationary phase (∼4.5 h), which is consistent with the data shown in Figure 2 and Figure 3. A similar expression pattern was obtained with the pPintC-gfp fusion for which the repression ratios were higher than for pPintS (28 in the exponential phase and 10 in the stationary phase). A high regulatory ratio was observed with the pPintF-gfp fusion whose expression was decreased around 23 times in the presence of the pIntF plasmid in exponential as well as in stationary growth phases, without any induction of the p*tac* promoter, indicating that the leak of the promoter allowed sufficient IntF production to produce a negative effect on the fusion expression. In contrast, the pPintL-gfp fusion was only down-regulated by a factor of 4 in the exponential growth phase, and this occurred in the presence of 0.1 mM of IPTG. Thus, the level of IntL required to lead to a negative effect on the fusion expression is probably higher than that necessary for the IntF integrase. One possible explanation is that integrase genes from *E. coli* MG1655 interfere with the downregulation of EDL933 genes. This hypothesis is strengthened by the fact that the regulatory ratios measured with the pPintC fusion were higher in an *intS* background than in a WT MG1655 background (data not shown). Together, these results supported the *in silico* prediction that STI and OD shape associated integrase genes should be negatively autoregulated. However, this prediction could be associated with promoter and recombination region sequence analysis to ensure that the two overlap.

**Table 1:** Strains and plasmids.

	Characteristics	References
**Strains**		
BW25113	*rrnB3* Δ*lacZ4787 hsdR514* Δ(*araBAD*)*567* Δ(*rhaBAD*)*568 rph-1*	[56]
ENZ1734	MG1655 Δ*lacIZ*	[55]
JW2345	BW25113 *intS*::Kan^R^	[56]
JW5383	BW25113 *tfaS*::Kan^R^	[56]
JW0525	BW25113 *intD*::Kan^R^	[56]
JW0275	BW25113 *intF*::Kan^R^	[56]
LCB1019	MC4100 ΔKplE1	[26]
LCB1024	MC4100 *intS* (Cm^s^)	[26]
LCB6005	ENZ1734 *tfaS*::Kan^R^	This work
LCB6006	ENZ1734 *intS*::Kan^R^	This work
LCB6007	ENZ1734 *intS* (Kan^S^)	This work
LCB6035	MC4100 *intD*::Kan^R^	This work
LCB6036	MG1655 *intF*::Kan^R^	This work
LCB6037	MC4100 *intD* (Kan^S^)	This work
LCB6038	MG1655 *intF* (Kan^S^)	This work
MG1655	F-*rfb-50 rph-1 ilvG*	M. Cashel
**Plasmids**		
pJF119EH	vector containing the p*_tac_* promoter with a *colE1* origin	[64]
pJFi	pJF119EH containing *torI* coding sequence	[59]
pBAD33	vector containing the p_ara_ promoter with a p*ACYC* origin	[38]
pBADintS	pBAD33 containing *intS* coding sequence	This work
pUA66	*gfpmut2* fusion vector with a p*sc101* origin	[37]
placZ-GFP	pUA66 containing the *lacZ* promoter region	[37]
pattL-gfp(wt)	pUA66 containing P*intS* (positions −223 to +64 relative to the ATG)	This work
pattL-gfp(O*) a	core mutated pattL-gfp	This work
pattL-gfp(I1*;2*) a	I1, I2 mutated pattL-gfp	This work
pattL-gfp(I3*;4*;5*) a	I3, I4, I5 mutated pattL-gfp	This work
pattL-gfp(I3*) a	I3 mutated pattL-gfp	This work
pattL-gfp(I4*) a	I4 mutated pattL-gfp	This work
pattL-gfp(I5*) a	I5 mutated pattL-gfp	This work
pattL-gfp(P'1*)^ a-^	P'1 mutated pattL-gfp	This work
pattL-gfp(P'1-A) a	P'1-A mutated pattL-gfp	This work
pattL-gfp (P'1-A) a	P'1-A mutated pattL-gfp	This work
pattL-gfp (P'1-A) a	P'1-A mutated pattL-gfp	This work
pattL-gfp(P'2*) a	P'2* mutated pattL-gfp	This work
pattL-gfp(P'3*) a	P'3* mutated pattL-gfp	This work
pattL-gfp(P'4*) a	P'4* mutated pattL-gfp	This work
pJFintC	pJF119EH containing *intC* (O157:7 EDL933) coding sequence	This work
pJFintD	pJF119EH containing *intD* (MG1655) coding sequence	This work
pJFintF	pJF119EH containing *intF* (MG1655)coding sequence	This work
pJFintH	pJF119EH containing *intH* (O157:7 EDL933) coding sequence	This work
pJFintL	pJF119EH containing *intL* (O157:7 EDL933) coding sequence	This work
pJFintS	pJF119EH containing *intS* (MG1655) coding sequence	This work
pPintC-gfp	pUA66 containing P*intC* (positions −205 to +81 relative to the ATG)	This work
pPintD-gfp	pUA66 containing P*intD* (positions −272 to +57 relative to the ATG)	This work
pPintF-gfp	pUA66 containing P*intF* (positions −145 to +71 relative to the ATG)	This work
pPintH-gfp	pUA66 containing P*intH* (positions −550 to +72 relative to the ATG)	This work
pPintL-gfp	pUA66 containing P*intL* (positions −273 to +70 relative to the ATG)	This work
pCP20	plasmid with temperature-sensitive replication and thermal induction of FLP synthesis	[57]

a mutations of the pattL-gfp plasmids are indicated in Figure 2C.

## Discussion

In temperate phages, site-specific recombination is a highly regulated process; indeed, both the activity and integrase gene expression are controlled. Little is known about integrase gene expression in general, except for the lambda phage integrase for which extensive studies have been available for almost 40 years. Integrase gene expression has been detected in natural environment samples induced with mitomycin C, which promotes productive growth. Therefore, integrase gene expression is used as a marker of (pro)phage presence [42].

In the case of the well-characterized lambda integrase, little integrase is made during lysogeny as none of the promoters is activated (*P_L_* and *P_I_*). Under lytic conditions, the *int* gene is transcribed together with the *xis* gene from the *P_L_* promoter due to the antitermination role of the N protein [1]. In KplE1, and other phages related by their recombination module such as HK620, Sf6 and CUS-3, transcription of the integrase and RDF genes is clearly uncoupled. Uncoupling of the integrase and RDF gene transcription has been described in P2 and 186 phages, where the *int* gene is expressed from the lysogenic transcript, and the RDF is the first gene on the lytic transcript [43]. The *intS* promoter, according to its orientation, overlaps with the *att*L region, where recombination proteins, including IntS itself, bind. We measured *intS* transcription during the *E. coli* exponential growth phase, and as expected, the *intS* transcript could be detected by RT-QPCR at the different time points of the growth ([26] and data not shown). In this work, we show that IntS as well as the RDF TorI negatively regulate *intS* expression. A similar situation is found in the P4 satellite phage, although the regulatory mechanism might be slightly different. In P4, Piazzolla and co-workers showed that the integrase and the RDF protein Vis negatively regulate *int* gene expression [44]. Although integrase self-regulation occurs through direct DNA binding at similar positions relative to the *int* transcription start site compared to those we described for IntS, the authors suggest that Vis binds to the *int* mRNA and therefore may inhibit translation [44]. In Figure 3, we show that TorI binds to DNA at positions favorable for transcription inhibition. In both cases, the RDF protein eventually promotes a lower integrase amount in the cell although Vis binds to RNA and TorI to DNA. These are the only documented cases of such a double down-regulation of integrase gene expression by the integrase itself and its cognate RDF protein. However, we can speculate that this regulatory process will be present in all cases where the integrase promoter overlaps the *att*L recombination region as long as the *att* region and integrase promoter overlap.

We then asked about the biological significance of such tight regulation of the integrase gene in KplE1. We measured the excisive reaction efficiency using fixed TorI and IHF concentrations, and variable IntS concentrations (Figure 4). Interestingly, the IntS concentration range that led to more than 50% efficiency was narrow, indicating that excisive recombination occurs at a precise integrase concentration. Moreover, when IntS was artificially overexpressed *in vivo*, the excision efficiency dropped rapidly as the IntS concentration increased (Figure 4). According to these results, a tight regulation of the integrase gene appears crucial for the recombination event to take place, as described earlier *in vivo* for lambda prophage [45]. The regulatory scenario characterized in KplE1 is dual. First, the integrase itself regulates its own expression by directly binding to the promoter sequence close to the −10 box. Negative autoregulatory loops are widespread in all organisms. For example, in mammals and photosynthetic bacteria, circadian oscillations are generated by a set of genes forming a transcriptional autoregulatory feedback loop [46], [47]. Feedback regulation plays a crucial role in the robust control of many cellular systems and is a way of stabilizing and maintaining the concentration of gene products. Recent models of feedback loops suggest that the strength of a feedback loop controls the oscillations of a regulatory path [48]. Therefore, one must consider the synthesis rate together with the degradation rate of the feedback regulator. In the case of IntS, the protein was very stable in the conditions we examined, suggesting that the loop is controlled only by the synthesis rate of the integrase. However, under certain conditions the integrase might undergo degradation by a yet unknown mechanism.

The second component of *intS* gene downregulation involves the TorI RDF (Figure 3). As mentioned above, this negative regulation involves the I4 and I5 sites located near the −35 sequence (Figure 2C). It is therefore likely that TorI prevents the binding of the RNA polymerase holoenzyme to the −35 region. This is a way to control the ratio integrase/RDF in order to obtain optimal excision conditions. In the lambda phage, the coupling of *xis* and *int* transcription upon lytic induction together with the presence of the *sib* untranslated region allow the accumulation of higher amounts of Xis than Int under lytic conditions [1], [34]. We therefore propose that the downregulation of *intS* by TorI is a different method of achieving a similar pattern in recombination protein concentrations. Indeed, when the prophage undergoes excision of its genome under lytic conditions, the RDF is needed in higher amounts than the integrase because of its dual role, directing the reaction towards excision and preventing the re-integration of the newly replicated phage genomes. As a consequence, in the absence of transcriptional coupling, as is the case in P4, KplE1 and other related (pro)phages, the RDF protein may directly control the appropriate integrase/RDF ratio through negative regulation of the integrase gene. Several lines of evidence support this statement; in particular, we showed in previous papers that the chromosomal *intS* gene was transcribed at a low level during the exponential growth phase [26] and that expressing the *torI* gene from a multicopy plasmid was sufficient to promote *in vivo* excision [25]. Altogether, our results show that the integrase gene is permanently expressed at a low level due to a strong negative control by the integrase itself and by the RDF. However, the gene is expressed at a sufficient level to allow prophage excision as soon as the RDF is produced [25]. Therefore, we propose that the main control of prophage excision targets the RDF gene when the integrase promoter is not coupled to the lytic promoter.

The narrow optimum Int concentration for recombination is probably the consequence of the strict stoechiometry required for the correct assembly of the intasome [12]. The role of the RDF protein is often restricted to a helper function as a DNA bender required to position the integrase molecules. However, the strict dependency of the lambda and KplE1 recombination systems on their respective RDF may suggest a more active role. In KplE1, the efficiency of the excisive recombination is dependent on the IntS/TorI protein ratio [13]. We speculate that, in this case, keeping a constant rate of integrase synthesis allows control of this ratio, and therefore the intasome forms only through the RDF production. Alternatively, researchers recently suggested that the alternative sigma factor (σ^H^) in *Staphylococcus aureus* participates in maintaining prophages by controlling integrase expression to ensure that more integrase than excisionase is made thus avoiding undesired excision [49]. Although *xis* transcription is strictly repressed by CI in the lambda prophage during lysogeny, one could imagine that transcriptional leakage is possible; thus, a moderate expression of the *int* gene could maintain the right balance of Int/Xis during lysogeny.

To address the general relevance of the negative autoregulation of the *intS* integrase gene, we performed a large-scale study of tRNA inserted prophages on complete prokaryotic genomes. The first step consisted of identifying of the *InTr* shapes. Current computational methods (Phage_Finder, Prophage Finder, DRAD) detect prophages in genomes by identifying possible essential proteins such as integrases, a region containing proteins similar to those occurring in prophages, or by dinucleotide relative abundance difference (DRAD) [40], [50], [51]. While these programs have been shown independently to give reliable results, comparative analysis of prophages identified by these methods showed high heterogeneity with low overlapping results probably arising from the mosaic nature of the prophages [51]. Therefore, we preferred identification without any *a priori*, based on the presence of the essential integrase gene. Moreover, our procedure can likely identify complete integrated elements and defective prophage regions encountered within prokaryotic genomes as long as they contain an integrase gene. The obtained data combined with tRNA searches gave 92.6% of STI *InTr* shapes in which the integrases have only a «Phage integrase» domain, and therefore, this procedure avoids many false positive results. Thus, without any *a priori* on the data, the most frequently observed STI shapes were with proteins likely to be similar to IntS, indicating a clear tRNA sublocation preference with this integrase subfamily. Several genome analysis studies showed that a vast majority of prophages are inserted in or adjacent to tRNA genes [39]–[41], [52], [53]. Williams revealed that tRNA sequence sites are preferred for prophage integration sites [39]. This analysis also demonstrated that for 34 cases out of 58 (59%) the *att*B sequence is in a tRNA or tmRNA and that some of the prophages are flanked by tRNA genes. A bias was also noted for the selenocysteinyl tRNA (tRNA SelC), tRNA Arg, tRNA Met, and tRNA Ala genes (Figure S2 and Table S3). The same conclusion was drawn by Fouts as his analysis of 285 putative attachment sites (from 302 complete bacterial genomes) revealed that tRNAs are the most frequently used targets (33%) for integration, and that the most popular tRNA targets are Arg, Leu, Ser and Thr [40]. Our integrase identification procedure, combined with the fact that we were working on >1000 organisms (compared to 302 bacterial genomes analyzed by Fouts [40]) may explain the difference observed (15% vs 33%) of the prophages that used tRNA as target sites. In *E. coli* and *Shigella* genomes, comparative genomic analysis also showed that tRNA loci are preferentially used as an insertion site for integrative elements, with the majority of tRNA genes remaining intact after insertion [52], [53]. Finally, Boyd and colleagues' analysis of island-encoded integrases revealed that half of the available tRNA genes were used as integration sites, in particular among members of the γ-Proteobacteria [41]. The vast majority of these integrase genes were adjacent to the tRNA loci. However, in the mentioned studies, less is done about functional relationships between the integrase and the proximal tRNA gene. We therefore focused on this particular couple of integration shapes, as some benefits could be expected by the genetic element from its association with a tRNA gene. As suggested by Swenson *et al*. a possible benefit could be the transcriptional coupling of the integrated element and the tRNA gene, as tRNA promoters are typically regulated by the growth rate [54]. Non-regularity in the orientation of prophages to tRNA genes has been observed, and researchers have suggested that the tRNA gene setting might directly affect integrase function or the directionality of recombination in a way that is beneficial for genetic elements.

The main focus of the *in silico* analysis was to study the occurrence of a regulatory path similar to the one we described for the *intS* gene. To our surprise, we found that the majority of tRNA associated integrase genes (61.5%) exhibited a promoter that overlapped with the *att*L recombination region (STI and OD shapes). As a consequence, and given the results we obtained with the *intS* promoter, we were able to predict that these genes may undergo negative autoregulation, which was confirmed *in vivo* for several genes (Figure 8). This prediction can be expanded to any locus containing an STI or OD *InTr* shape, as long as the recombination protein binding sites and RNA polymerase binding sites somehow overlap. For example, at the tRNA Ser locus in *Vibrio cholera*, the integrase gene associated to the genomic island VPI-2 should be autoregulated which may have some implication for the maintenance of this pathogenicity island.

### Concluding remarks

The regulatory switch leading to the controlled expression of the integrase and RDF proteins that allows the excision of the lambda prophage and therefore permits productive growth to resume has long been the paradigm for all temperate phages [1], [5]. In this study, we show that the particular organization we identified for the KplE1 *att*L recombination region and related (pro)phages is widespread among the tRNA inserted prophages. The fact that the *att*L region overlaps the integrase promoter has several consequences: (i) the integrase gene is likely down-regulated by itself and the RDF, as long as the recombination protein and the RNA polymerase binding sites overlap sufficiently, (ii) the transcription of the integrase and RDF genes are uncoupled, and (iii) the regulatory switch that permits prophage excision relies on RDF gene expression. Full understanding of prophage excision control will require focusing on the expression of the RDF genes that are uncoupled to the integrase gene transcription.

## Materials and Methods

### Bacterial strains, plasmids, media, and growth conditions

Bacterial strains and plasmids are listed in Table 1. Strains were grown in LB medium and, when necessary, ampicillin (50 µg mL^−1^), chloramphenicol (25 µg mL^−1^), kanamycin (25 µg mL^−1^) or IPTG (0.1–1 mM) were added.

### Strain construction

Strains LCB6005, LCB6006, LCB6035 and LCB6036 are derivatives of ENZ1734 (MG1655 Δ*lacIZ*) [55] obtained by P1 transduction of the *tfaS*::Kan^R^ (JW5383), *intS*::Kan^R^ (JW2345), *intD*::Kan^R^ (JW0525) and *intF*::Kan^R^ (JW0275) markers [56], respectively, into ENZ1734. The *kan* gene was then removed from strains LCB6006, LCB6035 and LCB6036 by using the pCP20 plasmid [57] to generate strains LCB6007 (*intS*, Kan^S^), LCB6037 (*intD*, Kan^S^), LCB6038 (*intF*, Kan^S^). Strains are described in Table 1.

### Plasmid construction

To construct plasmid pBintS, the *intS* coding sequence was PCR-amplified using MG1655 chromosomal DNA as a template with appropriate primers. After enzymatic hydrolysis, the PCR product was cloned into the KpnI/HindIII sites of the pBAD33 vector [38]. Plasmid pattL-gfp was constructed by the insertion of the *att*L region (220 bp, Figure 2C) into the XhoI and BamHI sites of the pUA66 vector [37]. A similar procedure was used to clone the promoter regions of *intD*, *intH*, *intL intF* into the pUA66 vector. Positions of the cloned sequences are indicated in Table 1, and primer sequences are available upon request from the authors. The sequence accuracy of the cloned inserts was checked by sequencing.

### Primer extension

Total RNAs extracted from strains MC4100 and LCB1024 (Δ*intS*), and strain LCB1019 (ΔKplE1) containing pattL-gfp were hybridized with a primer complementary to the positions +40 to +64 relative to the ATG of *intS* (attL-ter). attL-ter was ^32^P labeled by using [γ^32^P]ATP and T4 polynucleotide kinase (Biolabs). A total of 12 µg of ARNs and 4 ng of labeled primer were incubated together with 200 units of Superscript III reverse transcriptase (Invitrogen) for 50 minutes at 50°C, followed by 10 minutes at 70°C to inactivate the enzyme. The sequencing ladder was PCR amplified with the same labeled primer and 5′ primer hybridizing to positions −196 to −173 relative to the ATG of *intS* (attL-Kpn). The sequencing reaction was performed using the Thermo Sequenase Cycle Sequencing Kit (USB Corporation). Extension and sequencing products were separated onto a 6 M urea 8% acrylamide (19∶1) gel.

### Site-directed mutagenesis of *att*L

Mutations in the recombination protein binding sites were generated by an overlapping PCR procedure [58]. Mutated primers were used to amplify the protein binding sites whereas the wild-type primers attL-pro-XhoI and attL-ter-BamHI delimit the *attL* region. After enzymatic hydrolysis, mutated *att*L were cloned into pUA66. Mutations in the IntS and TorI binding site are summarized in Figure 2C. All primer sequences used for mutagenesis are available upon request.

### Protein purifications

IntS, TorI and IHF proteins were overproduced and purified near homogeneity as described [26], [59], [60]. All proteins were dialyzed in 40 mM Tris-HCl buffer (pH 7.6) containing 50 mM KCl and 10% glycerol. Denaturing polyacrylamide gel electrophoresis (SDS-PAGE) was used to estimate the protein purity, and the Lowry method was used to estimate protein concentrations.

### *In vivo* excision assay

Strain LCB6005 (Kan^R^ gene inserted in the *tfaS* gene of KplE1) carrying plasmids pJFi and pBAD33 (control) or pJFi and pBintS were grown in LB medium supplemented by increasing amounts of arabinose as indicated in Figure 5 legend. When the A_600_ reached 0.5 units (0.5×10^9^ cells mL^−1^), IPTG (1 mM) was added and the culture resumed for 2 h at 37°C under agitation. Culture dilutions were prepared and plated onto rich medium containing either ampicillin (pJFi) or kanamycin (*tfaS::kan*). Numeration of the colonies plated on both antibiotics was performed and the ratio of ampicillin-resistant/kanamycin-resistant (Ap^R^/Kn^R^) colonies was calculated. This value is close to one when the excision rate is low and the *tfaS::kan* marker is present, and increases when excision efficiency increases and the cells no longer contain the KplE1 prophage. The values represent the average of at least three independent determinations. The IntS relative amount in crude extracts was analyzed after 12% SDS-PAGE with Western blot using a polyclonal IntS antiserum.

### *In vitro* excision assay

Purified IHF, IntS and TorI were used in all experiments. All reaction mixtures (25 µl) included 32 nM of linear *att*L (attL-SpeI/attL-KpnI primers) and *att*R (attR-XbaI/attR-IHF2 primers) in buffer containing 30 mM Tris-HCl (pH 7.6), 10 mM spermidine, 5 mM EDTA, 1 mg.mL^−1^ bovine serum albumin, 34 mM KCl and 5% glycerol. IHF (0.25 µM) and TorI (1.6 µM) were added in all samples in the presence of a range of IntS concentrations (0.02 to 8 µM). The reactions were carried out in optimized conditions: 37°C for 1 h. The best efficiency was obtained for IntS concentrations ranging from 0.8 to 1.2 µM, leading to an IHF:IntS:TorI protein ratio of 1∶4∶6.

### Real-time PCR analysis (Q-PCR)

The abundance of *att*P formed during *in vitro* excision assays was quantified by real-time PCR, using a known concentration of PCR amplified *att*P as a reference standard. The real-time PCR quantifications were performed with an Eppendorf Mastercycler ep realplex instrument and the SYBR *Premix Ex Taq* (TaKaRa) according to the manufacturer specifications. Serial dilutions of each *in vitro* reaction were mixed with 1.5 µM of primers and 6 µL of master mix in a 14 µL final volume. The primer pair used to quantify *att*P was attR-IHF2/attL-SpeI. PCR parameters were as follows: one cycle at 95°C for 2 min followed by 40 cycles at 95°C for 5 s, 55°C for 15 s and 72°C for 10 s. Excision efficiency was calculated as the percentage of the initial substrate (32 nM) transformed into product.

### GFP transcriptional fusion measurement

GFP fluorescence was measured on whole cells after an overnight aerobic growth at 37°C in LB medium supplemented by IPTG (0.1–1 mM) for TorI and/or integrase induction (Figure 3). The pJF119EH empty vector was used as a negative control and to ensure that the growth conditions (presence of ampicillin) were identical for all strains. After centrifugation, bacteria were washed, resuspended and diluted in 0.25X M9 medium. Cells (150 µL) were loaded on an Optilux black/clear Bottom Microtest 96-well assay plate (Falcon). Alternatively, fluorescence intensity was measured on bacterial cultures over time. Precultures of the various strains were diluted in fresh LB medium containing the appropriate antibiotics and IPTG (0.1 mM) when indicated. Each strain was assayed in quadruplet. The incubation protocol included an initial 5-min shake (double orbital, 1.5 mm diameter, normal speed), followed by 85 cycles consisting of the following actions: a 1-sec measurement (see below), a 6-min shake and a 1-min standing. The time course was performed at 37°C for approximately 10 h. A_600_ and fluorescence measurements were performed using the Infinit M200 instrument (Tecan) and the Tecan i-control 1.3 application (488 nm excitation wavelength, 521 nm emission wavelength, 160 gain, 20 µs integration time and 25 reads per sample). The value of blank (0.25X M9 or LB) was withdrawn and normalized fluorescence intensities (emission at 521 nm/A_600_) were calculated. The values represent the averages of at least four independent measurements. Microscopic analysis was performed using an automated and inverted epifluorescence microscope TE2000-E-PFS (Nikon, France) and adequate filters (excitation 480±15 nm, emission 535±20 nm). Images were recorded with a CoolSNAP HQ 2 (Roper Scientific, Roper Scientific SARL, France) and a 40x/0.75 DLL “Plan-Apochromat” or a 100x/1.4 DLL objective; image analysis was conducted with MetaMorph 7.5 software (Molecular Devices). For each cell preparation, a total of 25 images were taken randomly on different optical fields, and the average intensity of each cell was calculated (Figure S1).

### Bioinformatic analyses

The complete genomes of 1014 prokaryotic (946 bacterial and 68 archaeal) organisms available in December 2009 were downloaded from the NCBI ftp site (ftp:/ftp.ncbi.nih.gov/genomes/Bacteria/) and constitute the primary data source. To identify integrase promoters overlapping the integration site, the analysis was restricted to prophage insertion targeted to tRNA sites. The HMMER-3 package [61] and self-written Perl scripts were then used to search for protein integrase homologs (with phage λ *int* and *E. coli intS* as reference seed proteins) in the complete genomes. The presence of one of these functional domains (from Pfam 24.0 [62]), Phage_integrase (PF00589), Phage_integ_N (PF09003) or Phage_integr_N (PF02899), was a requisite. Alignments with a score higher than the Pfam gathering thresholds were considered significant. Note that homologs with protein sizes lower than 140 amino acids (corresponding to 80% of the Phage_integrase profile length) were removed from the data. The obtained sequences were subsequently analyzed with the same software in order to locate additional known functional domains. In-house Perl scripts were used to define the domain organization. The search for tRNA genes, located in the region between the integrase gene and the downstream/upstream neighboring gene was performed by using the tRNAscan-SE program [63]. Finally, protein integrase homologs were filtered by the presence of an adjacent tRNA gene (downstream or upstream of the integrase gene), leading to the final set of integrase homologs used in this study. We then computed the IR length as the distance in nucleotides between a given integrase gene and the immediately adjacent tRNA gene.

## Supporting Information

Figure S1 Expression of the *attL-gfp* transcriptional fusion in the bacterial population. LCB6007 (*ΔintS*)/pJF119EH (0), LCB6007/pJFi (+TorI) or ENZ1734 (wt)/pJF119EH (+IntS) strains were transformed with pUA66-gfp (empty vector, A.) and pattL-gfp (wt, B.). After an overnight aerobic growth in the presence of 1 mM of IPTG for *torI* induction, the average fluorescence of the bacteria was calculated (see the Materials and Methods). Population distributions according to the average fluorescence are plotted.
(0.22 MB TIF)

Figure S2 *InTr* insertion biases with respect to the tRNA codon. For each tRNA, the *InTr* tRNA codon bias was computed as Obs/All where Obs, is the proportion of *InTr* tRNA codon shapes over the total number of *InTr* shapes and All is the proportion of the same *InTr* shape codon over the total number of tRNA codons in the 561 organisms. Threshold ratios for positive and negative biases are set to [1] and [−1], respectively. For more details, see Table S1.
(0.16 MB TIF)

Table S1 A detailed description of all tRNA associated integrase genes present in prokaryotic genomes that constitute the primary data set used in this study.
(0.49 MB XLS)

Table S2 The integrase insertion bias in close proximity of each tRNA was calculated as *Obs/Exp* where Obs is the proportion of specific *InTr* shapes (over the 1273 *InTr* shapes) and Exp, the proportion of the same tRNA out of the overall tRNA in 561 genomes. If the ratio *Obs/Exp* is <1, the bias becomes -*Exp/Obs.* Note that Pseudo, Sup and Undef tRNAs (291 tRNAs from a total of 34596) were removed from our data. %AR, is the proportion of predicted autoregulated *InTr* shapes. Note that in four cases, the *InTr* shapes were found within the plasmids eg. 2 in *Silici bacter*_TM140 (NC008043, Ser-OC and Phe-TI), 1 in *Ralstonia eutropha* JM134 (NC_007336, Met-TI) and 1 in *Burkholderia phymatum* STM 185 (NC_010625, Leu-OC).
(0.05 MB DOC)

Table S3 For each tRNA, the *InTr* tRNA codon bias was computed as Obs/All, where Obs is the proportion of *InTr* tRNA codon shapes over the total number of *InTr* shapes and All is the proportion of the same *InTr* shape codon over the total number of tRNA codons in the 561 organisms. Threshold ratios for positive and negative biases are [1] and [−1], respectively. One hundred and six uncertain codons, one TAA codon and two TAG codons (from Sup tRNA) were removed from the data (34887 codons from the 561 genomes with *InTr* shapes). <10–4, less than 0.0001. Negative and positive biases are marked by (−) and (+), respectively.
(0.05 MB DOC)

In silico analysis of tRNA associated integrase genes in prokaryotic genomes. The supporting text contains a detailed analysis of tRNA associated genes in prokaryotic genomes.
(0.39 MB DOCX)
