# Supplementary figures and images for: Tight Regulation of the intS Gene of the KplE1 Prophage: A New Paradigm for Integrase Gene Regulation
Source: PLoS Genet. 2010 Oct 7;6(10):e1001149. doi: 10.1371/journal.pgen.1001149 (PMC2951348; doi:10.1371/journal.pgen.1001149)

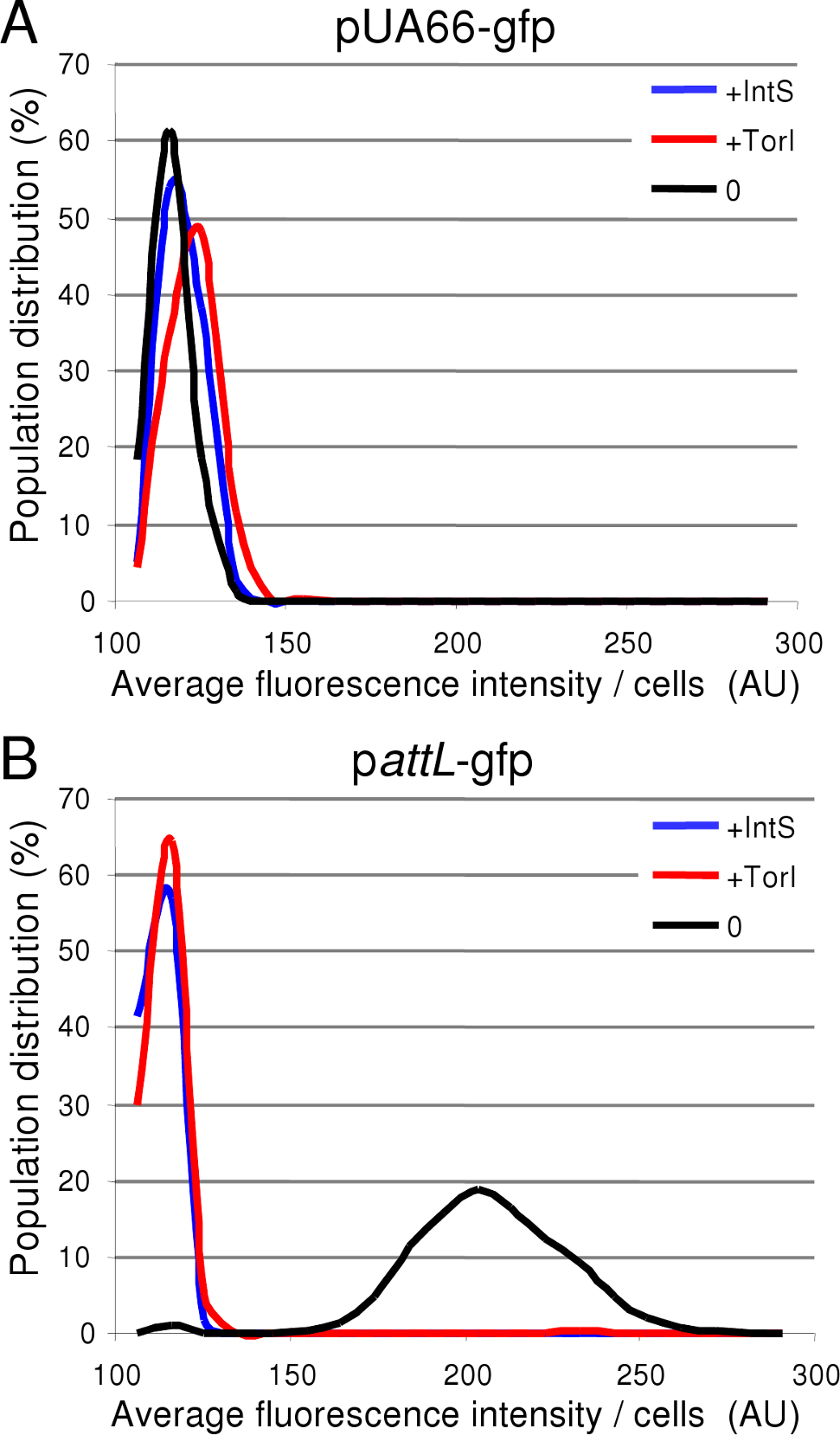

Supplement: Figure S1 — Expression of the attL-gfp transcriptional fusion in the bacterial population. LCB6007 (ΔintS)/pJF119EH (0), LCB6007/pJFi (+TorI) or ENZ1734 (wt)/pJF119EH (+IntS) strains were transformed with pUA66-gfp (empty vector, A.) and pattL-gfp (wt, B.). After an overnight aerobic growth in the presence of 1 mM of IPTG for torI induction, the average fluorescence of the bacteria was calculated (see the Materials and Methods). Population distributions according to the average fluorescence are plotted. (0.22 MB TIF) [file pgen.1001149.s001.tif]

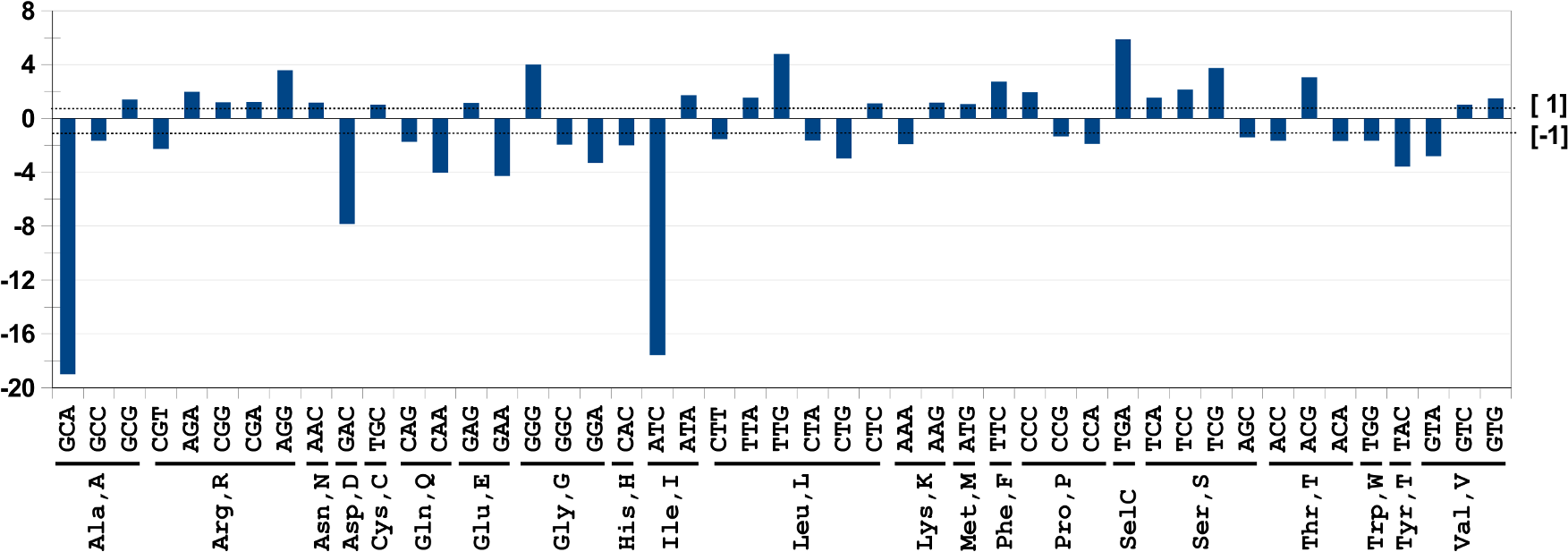

Supplement: Figure S2 — InTr insertion biases with respect to the tRNA codon. For each tRNA, the InTr tRNA codon bias was computed as Obs/All where Obs, is the proportion of InTr tRNA codon shapes over the total number of InTr shapes and All is the proportion of the same InTr shape codon over the total number of tRNA codons in the 561 organisms. Threshold ratios for positive and negative biases are set to [1] and [−1], respectively. For more details, see Table S1. (0.16 MB TIF) [file pgen.1001149.s002.tif]
