# Supplementary material for: Tight Regulation of the intS Gene of the KplE1 Prophage: A New Paradigm for Integrase Gene Regulation
Source: PLoS Genet. 2010 Oct 7;6(10):e1001149. doi: 10.1371/journal.pgen.1001149 (PMC2951348; doi:10.1371/journal.pgen.1001149)
Supplement: Table S2 — The integrase insertion bias in close proximity of each tRNA was calculated as Obs/Exp where Obs is the proportion of specific InTr shapes (over the 1273 InTr shapes) and Exp, the proportion of the same tRNA out of the overall tRNA in 561 genomes. If the ratio Obs/Exp is <1, the bias becomes -Exp/Obs. Note that Pseudo, Sup and Undef tRNAs (291 tRNAs from a total of 34596) were removed from our data. %AR, is the proportion of predicted autoregulated InTr shapes. Note that in four cases, the InTr shapes were found within the plasmids eg. 2 in Silici bacter_TM140 (NC008043, Ser-OC and Phe-TI), 1 in Ralstonia eutropha JM134 (NC_007336, Met-TI) and 1 in Burkholderia phymatum STM 185 (NC_010625, Leu-OC). (0.05 MB DOC) [file pgen.1001149.s004.doc]

**Table S2.** Distribution of *InTr* shapes with respect to tRNA.

|  | ***STI*** | ***SIT*** | ***OC*** | ***OD*** | ***InTr*** | ***% InTr (Obs)*** | ***Biais (Obs/Exp)*** | ***%AR*** |
| --- | --- | --- | --- | --- | --- | --- | --- | --- |
| ***Ala, A*** | 17 | 1 | 13 | 0 | 31 | 2.44 | -2.94 | 54.8 |
| ***Arg, R*** | 69 | 3 | 84 | 8 | 164 | 12.88 | 1.49 | 46.9 |
| ***Asn,N*** | 46 | 0 | 4 | 3 | 53 | 4.16 | 1.19 | 92.4 |
| ***Asp, D*** | 4 | 0 | 2 | 0 | 6 | 0.47 | -7.69 | 66.7 |
| ***Cys, C*** | 18 | 2 | 4 | 1 | 25 | 1,96 | 1.04 | 76.0 |
| ***Gln, Q*** | 14 | 0 | 2 | 0 | 16 | 1.26 | -2.85 | 87.5 |
| ***Glu, E*** | 8 | 0 | 14 | 0 | 22 | 1.73 | -1.70 | 36.4 |
| ***Gly, G*** | 69 | 11 | 12 | 3 | 95 | 7.46 | 1.03 | 75.8 |
| ***His, H*** | 8 | 1 | 3 | 0 | 12 | 0.94 | -2.0 | 66.7 |
| ***Ile, I*** | 1 | 1 | 2 | 0 | 4 | 0.31 | -109.9 | 25.0 |
| ***Leu, L*** | 126 | 5 | 51 | 3 | 185 | 14.53 | 1,5 | 69.7 |
| ***Lys, K*** | 11 | 6 | 25 | 1 | 43 | 3.38 | -1.45 | 27.9 |
| ***Met, M*** | 52 | 9 | 37 | 9 | 107 | 8.41 | 1.07 | 57.0 |
| ***Phe, F*** | 62 | 1 | 21 | 1 | 85 | 6.68 | 2.77 | 74.1 |
| ***Pro, P*** | 27 | 3 | 24 | 5 | 59 | 4.63 | -1.01 | 54.2 |
| ***SelC*** | 35 | 0 | 2 | 0 | 37 | 2.92 | 5.85 | 94.6 |
| ***Ser, S*** | 95 | 6 | 59 | 6 | 166 | 13.04 | 1.88 | 60.8 |
| ***Thr, T*** | 39 | 1 | 37 | 6 | 83 | 6.52 | 1.16 | 54.2 |
| ***Trp, W*** | 8 | 0 | 6 | 0 | 14 | 1.1 | -1.64 | 57.1 |
| ***Tyr, T*** | 2 | 2 | 5 | 0 | 9 | 0.71 | -3.57 | 22.2 |
| ***Val, V*** | 25 | 0 | 31 | 1 | 57 | 4.48 | -1.40 | 45.6 |
| ***Overall*** | **736** | **52** | **438** | **47** | **1273** | **100** | **-** | **61.5** |
