# Supplementary material for: Tight Regulation of the intS Gene of the KplE1 Prophage: A New Paradigm for Integrase Gene Regulation
Source: PLoS Genet. 2010 Oct 7;6(10):e1001149. doi: 10.1371/journal.pgen.1001149 (PMC2951348; doi:10.1371/journal.pgen.1001149)
Supplement: Table S3 — For each tRNA, the InTr tRNA codon bias was computed as Obs/All, where Obs is the proportion of InTr tRNA codon shapes over the total number of InTr shapes and All is the proportion of the same InTr shape codon over the total number of tRNA codons in the 561 organisms. Threshold ratios for positive and negative biases are [1] and [−1], respectively. One hundred and six uncertain codons, one TAA codon and two TAG codons (from Sup tRNA) were removed from the data (34887 codons from the 561 genomes with InTr shapes). <10–4, less than 0.0001. Negative and positive biases are marked by (−) and (+), respectively. (0.05 MB DOC) [file pgen.1001149.s005.doc]

**Table S3.** *InTr* codon shape frequencies and biases.

| ***tRNA*** | ***Codon*** | **% obs** | **% 561** | **Biais** | ***tRNA*** | ***Codon*** | **% Obs** | **% 516** | **Biais** |
| --- | --- | --- | --- | --- | --- | --- | --- | --- | --- |
| ***Ala, A*** | **GCT**  **GCC**  **GCA**  **GCG** | -  1.18  0.24  1,02 | -  1.96  4.56  0.72 | -  -1.66  -19.0  1.41 | ***Lys, K*** | **AAA**  **AAG** | 1.96  1.41 | 3.74  1.19 | -1.91  1.18 |
| ***Arg, R*** | **CGT**  **CGC**  **CGA**  **CGG**  **AGA**  **AGG** | 1.41  -  0.47  1.81  4.01  5.18 | 3.18  0.14  0.39  1.50  2.01  1.45 | -2.26  -  1.21  1.21  1.99  3.58 | ***Met, M*** | **ATG** | 8.41 | 7.88 | 1.07 |
| ***Asn, N*** | **AAT**  **AAC** | -  4.16 | -  3.49 | -  1.19 | ***Phe, F*** | **TTT**  **TTC** | -  6.68 | <10^-4^  2.43 | -  2.75 |
| ***Asp, D*** | **GAT**  **GAC** | -  0.47 | -  3.69 | -  -7.84 | ***Pro, P*** | **CCT**  **CCC**  **CCA**  **CCG** | -  2.59  1.18  0.86 | <10^-4^  1.33  2.22  1.14 | -  1.95  -1.88  -1.33 |
| ***Cys, C*** | **TGT**  **TGC** | -  1.96 | -  1.91 | -  1.03 | ***SelC*** | **TGA** | 2.91 | 0.49 | 5.88 |
| ***Gln, Q*** | **CAA**  **CAG** | 0.63  0.63 | 2.54  1.09 | -4.03  -1.73 | ***Ser, S*** | **TCT**  **TCC**  **TCA**  **TCG**  **AGT**  **AGC** | -  4.01  3.22  4.56  -  1.26 | <10^-4^  1.85  2.10  1.21  -  1.77 | -  2.16  1.54  3.76  -  -1.41 |
| ***Glu, E*** | **GAA**  **GAG** | 0.94  0.79 | 4  0.68 | -4.25  1.16 | ***Thr, T*** | **ACT**  **ACC**  **ACA**  **ACG** | -  1.18  1.41  3.93 | 0.04  1.96  2.35  1.28 | -  -1.66  -1.67  3.06 |
| ***Gly, G*** | **GGT**  **GGC**  **GGA**  **GGG** | -  2.04  0.63  4.79 | -  3.97  2.09  1.2 | -  -1.95  -3.31  4.0 | ***Trp, W*** | **TGG** | 1.10 | 1.82 | -1.66 |
| ***His, H*** | **CAT**  **CAC** | -  0.94 | -  1.87 | -  -1.99 | ***Tyr, Y*** | **TAT**  **TAC** | -  0.71 | -  2.53 | -  -3.57 |
| ***Ile, I*** | **ATT**  **ATC**  **ATA** | -  0.24  0.08 | <10^-4^  4.21  0.05 | -  -17.56  1.74 | ***Val, V*** | **GTT**  **GTC**  **GTA**  **GTG** | -  2.04  1.26  1.18 | <10^-4^  1.96  3.52  0.79 | -  1.04  -2.79  1.49 |
| ***Leu, L*** | **TTA**  **TTG**  **CTT**  **CTC**  **CTA**  **CTG** | 2.91  7.62  0.08  1.89  1.26  0.79 | 1.89  1.59  0.12  1.67  2.05  2.35 | 1.54  4.80  -1.55  1.13  -1.63  -2.97 |  |  |  |  |  |
