# Supplementary material for: Tight Regulation of the intS Gene of the KplE1 Prophage: A New Paradigm for Integrase Gene Regulation
Source: PLoS Genet. 2010 Oct 7;6(10):e1001149. doi: 10.1371/journal.pgen.1001149 (PMC2951348; doi:10.1371/journal.pgen.1001149)
Supplement: Supplementary file 6 — In silico analysis of tRNA associated integrase genes in prokaryotic genomes. The supporting text contains a detailed analysis of tRNA associated genes in prokaryotic genomes. (0.39 MB DOCX) [file pgen.1001149.s006.docx]

Supporting Information

Supporting text:

*InTr* shapes are not uniformly distributed among prokaryotic genomes

To address the general relevance of the negative autoregulation of the *intS* integrase gene, a bioinformatic analysis of tRNA inserted prophages was performed in a large scale on the complete prokaryotic genomes available at NCBI. The *in silico* outline is described in the “Materials and Methods” section. Experimentally well characterized integrases such as ^λ^Int and ^KplE1^IntS contain at least one of the three functional domains: Phage_integrase, Phage_integ_N, and Phage_integr_N, referred as PF00589, PF09003 and PF02899 in Pfam database, respectively. By using these functional domains as queries, we detected 8368 protein homologs within 1014 complete prokaryotic genomes. Analysis of those putative prophage regions revealed that 1273 (15 % of the putative protein integrases) are in close proximity with a tRNA gene. These couples of tRNA-integrase genes (called *InTr* shape) constitute the primary data set used in this study (see Table S1). Interestingly, the PF00589 domain is present in all the 1273 proteins and a vast majority of proteins (92.6 %) harbored a single PF00589 domain. Additional domains (*e.g.* AP2, a DNA binding domain of 60 amino acids, PF00847; HTH_3, a DNA binding helix-turn-helix domain, PF01381; TraI_2, a putative helicase domain, PF07514; DEAD, a helicase domain, PF00270) are found in 28 proteins. The *InTr* shapes were present in 561 prokaryotic genomes (about 55 % of the analyzed organisms, Fig. 6) and were found in most of the prokaryotic phyla (19 out of 23 referenced taxonomic groups), with the exception of the Thermotogales, Aquificales, Nanoarcheota and Chorarcheota. However, striking differences are exhibited by the phyla. For example, only one genome (***Treponema denticola* str. ATCC 35405**) over the 18 Spirochaetes organisms was found to contain an *InTr* shape. The same observation can be drawn for the Cyanobacteria and Firmicutes phyla in which, only one-third of the analyzed genomes contains *InTr* shape representatives. Interestingly, *InTr* shapes are well represented in Archaeal bacteria (in particular in Crenarchaeota and Euryarchaeota). The group of Proteobacteria, with the largest number of completely sequenced genomes, also contains the highest number of *InTr* shapes, in particular in γ-Proteobacteria with more than two *InTr* per organism over the overall phylum. We further investigated the occurrence of *InTr* copies per organism (Fig. 6B). While the average number of *InTr* shape copy per genome is about 2.27, this figure shows heterogeneity of the *InTr* copy number per organism. A majority of organisms (248/561=44.2 %) harbors a single *InTr* copy within their genomes. This percentage is not uniformly distributed over the prokaryotic taxonomic groups. While 71 % to 90 % of the *InTr* identified in Firmicutes (e.g. in *Streptococcus* genus (18 strains) including *pyogenes*, *suis* and *agalactie* species), Cyanobacteria (*e.g*. the four *Prochlorococcus marinus* strains) or Euryarchaeota (e.g. the 3 *Methanococcus maripaludis* strains) are in one copy per genome, other phyla (*e.g*. δ-Proteobacteria, γ-Proteobacteria) mainly exhibit two or more *InTr* copies per organism. Interestingly, we found that closely related organisms may harbor different *InTr* shape copy numbers. For example, 3 to 5 *InTr* shape copies are found in *Yersinia pestis* genomes including subspecies *Angola*, *Antiqua*, *biovar-microtus-91001*, *CO92*, *KIM10*, *Nepal516*, and *Pestoides*, indicating that these genomes are not equivalent regarding prophage insertion and justifying the use of closely related genomes in this study. Analysis of the genomes of *Xanthomonas campestris vesicatoria 85-10*, *Methylobacterium nodulans ORS 2060* and *Pectobacterium wasabiae WPP163* show a high concentration of *InTr* shapes with 9 and 11 copies, respectively. Together these results clearly show that the number of *InTr* shapes is not uniformly distributed among the prokaryotic genomes indicating that the insertion within a tRNA gene is a strategy variously employed by temperate phages.

Prokaryotic phyla show preference for different *InTr* shapes

We analyzed the preference for a particular *InTr* shape in a given organism or a given prokaryotic phylum. Therefore, *InTr* shapes were classified according to their gene coding orientation, leading to four different types of *InTr* shapes (Fig. 6A): STI (Same orientation and T precedes I), SIT (Same orientation and I precedes T), OC (Opposite and Convergent orientation) and OD (Opposite and Divergent orientation). The overall analysis of the obtained data is presented in Figure 6C. STI shapes are very abundant in Crenarcheota (*e.g.* *Sulfolobus tokodaii* with 2 STI shapes), Bacteroidetes (*e.g.* *Bacteriodes fragilis YCH46* and *Bacteriodes fragilis NCTC 9434* strains with 5 and 4 STI shapes, respectively), Cyanobacteria (*e.g.* *Acaryochloris marina MBIC 11017* with 4 STI shapes), α-Proteobacteria, β-Proteobacteria and γ-Proteobacteria. OC couples were significantly found in Deinococcus (*e.g.* *Thermus thermophilus HB27*, 2 OC shapes), Acidobacteria (*e.g. Candidatus koribacter versatilis Ellin345*, 3 OC shapes), Firmicutes (e.g. *Lactobacillus casei*, 3 OC shapes), and Actinobacteria (*e.g*. *Streptomyces coelicolor*, 3 OC shapes).

Therefore, the majority of *InTr* shapes exhibits STI and OC shapes with 736 and 438 representatives, respectively. The other two classes (SIT and OD) are of relatively rare occurrence (less than 8 % in total) in the analyzed genomes. While in STI or OC examples described above only one specific type of *InTr* shapes is found within the same organism, different types of *InTr* shapes are found in a number of organisms. This was the case for *E. coli* strains (*e.g.* 1 STI, 1 OD and 1 OC shapes in subspecies *E24377A*; 2 STI, 1 OC and 1 OD shapes in *K-12 substr. DH10B* and *W3110*; and 4 STI, 2 OC and 1 OD shapes in *O157:H7 str. TW14359*). Finally, the high occurrence of STI and OC shapes within the prokaryotes may highlight the functional importance of these shapes in microbial organisms.

Correlation between *InTr* shape and the type of tRNA

In the next step, we analyzed the STI, SIT, OC and OD shapes regarding the type of tRNA (Table S2). The number of STI and OC shapes was higher for each tRNA, as a consequence of the higher occurrence of these *InTr* shapes in the analyzed organisms. Moreover, the occurrence of STI or OC shapes is significantly different with the type of tRNA. Indeed, the STI shape is preferentially observed for Ala, Asn, Cys, Gln, Gly, His, Leu, Met, Phe, SelC, and Ser tRNAs, whereas the OC shape is preferentially detected for Arg, Glu, Lys, Tyr, and Val. For the remaining tRNAs, we considered that there is no significant difference between the number of STI and OC shapes. Interestingly, Arg remains one of the most frequent tRNA gene associated with prophage regions in our analysis as previously described (Fouts, 2006). Note that in four cases, *InTr* shapes were found within plasmids (eg. 2 in *Silicibacter_TM140* (NC008043, Ser-OC and Phe-STI), 1 in *Ralstonia eutropha* JM134 (NC_007336, Met-STI) and 1 in *Burkholderia phymatum STM 185* (NC_010625, Leu-OC). Our analysis of the protein integrases revealed that less than 4 % (1273/34576) of the available tRNA genes were used as integration sites, in particular among members of the γ-Proteobacteria.

In order to determine whether the observed *InTr* shapes is dependent or not of the tRNA occurrence within the genomes, we compute for each tRNA the relative biases associated to prophage insertion (Table S2). Highly positive biases were then observed for SelC, Phe and Ser, suggesting that these tRNAs are respectively 5.85, 2.77, and 1.88 times more used as a target for phage insertion than what should be randomly expected by their occurrence in the genome. The opposite conclusion can be drawn for Ile, Asp, and Tyr with negative biases of 109.9, 7.69 and 3.57, respectively. We next assessed phage insertion with respect to the tRNA codon (Table S3). For each tRNA, there is a prophage insertion preference for a tRNA codon (e.g. Arg AGA and AGG, Gly GGG, or Leu TTG). The observed insertion rate of tRNA codon exhibits positive biases (e.g. SelC TGA, Leu TTG and Ser TCG codons) and negative biases (e.g. Ile ATC, Ala GCA and Asp GAC). Overall, our analysis showed that prophage insertions toward the tRNA insertion site is subjected to high variation with respect to tRNA and tRNA codon types.

Discussion

In our study, we noticed an insertion preference for the Leu, Ser and Arg tRNAs, result that is in complete agreement with Fouts (Fouts, 2006). However, different results were obtained by the Williams analysis for which a bias was noted for the SelC, Arg, Met and Ala tRNA genes (Williams, 2002). Interestingly, insertions adjacent to polycystronic tRNA loci (at least 2 tRNA genes) are found in 11.7% (149/1273) of *InTr* with most of them having Leu tRNA genes as the neighbor of the protein integrase (see supplemental Table 2). These particular *InTr* spanned several microbial phyla including Firmicutes (e.g. *Bacillus cereus*, 16 tRNAs genes), Fusobacteria (e.g. *Streptobacillus* *monoliformis DSM12112*, 9 tRNAs), γ-Proteobacteria (e.g. *Aeromonas salmonicida A449*, 8 tRNAs), Euryarchaeota (e.g. *Methanococcus aeolicus Nankai-3*, 6 tRNAs), or Actinobacteria (e.g. *Mycobacterium ulcerans Agy99*, 4 tRNAs). Our analysis also revealed that the insertion preference (based on the absolute number of InTr shapes) does not correlate with the statistical biases taking into account the actual proportion of tRNA within the analyzed genomes. This new factor introduces the concept of positive and negative tendency facing the phage insertion in tRNA insertion sites, when statistical biases have positive and negative values, respectively.

Our results also revealed the occurrence of varied types of *InTr* shapes in diverse group of prokaryotes. Furthermore, different bacterial phyla show preference for different types of STI couples and this propensity is correlated with autoregulation of the integrase gene. Thus, it seems that integrases have evolved specificity to certain tRNA genes and this specificity can be taxa specific, indicating a long evolutionary relationship between integrases and their insertion sites. The exploration of this large diversity of tRNA site integration revealed that most tRNA genes are subjected to phage integration with some preference with certain tRNA genes. This unlimited number of tRNA gene or specific tRNA codon suggested a random insertion of the phage. Boyd and colleagues suggested that integration into tRNAs that are highly expressed is probably avoided because this, too, could have serious fitness effects for the cell (Boyd et al., 2009).

**Table S1** (separated file)

**Table S2.** Distribution of *InTr* shapes with respect to tRNA.

|  | ***STI*** | ***SIT*** | ***OC*** | ***OD*** | ***InTr*** | ***% InTr (Obs)*** | ***Biais (Obs/Exp)*** | ***%AR*** | |
| --- | --- | --- | --- | --- | --- | --- | --- | --- | --- |
| ***Ala, A*** | 17 | 1 | 13 | 0 | 31 | 2.44 | -2.94 | 54.8 |  |
| ***Arg, R*** | 69 | 3 | 84 | 8 | 164 | 12.88 | 1.49 | 46.9 |  |
| ***Asn,N*** | 46 | 0 | 4 | 3 | 53 | 4.16 | 1.19 | 92.4 |  |
| ***Asp, D*** | 4 | 0 | 2 | 0 | 6 | 0.47 | -7.69 | 66.7 |  |
| ***Cys, C*** | 18 | 2 | 4 | 1 | 25 | 1,96 | 1.04 | 76.0 |  |
| ***Gln, Q*** | 14 | 0 | 2 | 0 | 16 | 1.26 | -2.85 | 87.5 |  |
| ***Glu, E*** | 8 | 0 | 14 | 0 | 22 | 1.73 | -1.70 | 36.4 |  |
| ***Gly, G*** | 69 | 11 | 12 | 3 | 95 | 7.46 | 1.03 | 75.8 |  |
| ***His, H*** | 8 | 1 | 3 | 0 | 12 | 0.94 | -2.0 | 66.7 |  |
| ***Ile, I*** | 1 | 1 | 2 | 0 | 4 | 0.31 | -109.9 | 25.0 |  |
| ***Leu, L*** | 126 | 5 | 51 | 3 | 185 | 14.53 | 1,5 | 69.7 |  |
| ***Lys, K*** | 11 | 6 | 25 | 1 | 43 | 3.38 | -1.45 | 27.9 |  |
| ***Met, M*** | 52 | 9 | 37 | 9 | 107 | 8.41 | 1.07 | 57.0 |  |
| ***Phe, F*** | 62 | 1 | 21 | 1 | 85 | 6.68 | 2.77 | 74.1 |  |
| ***Pro, P*** | 27 | 3 | 24 | 5 | 59 | 4.63 | -1.01 | 54.2 |  |
| ***SelC*** | 35 | 0 | 2 | 0 | 37 | 2.92 | 5.85 | 94.6 |  |
| ***Ser, S*** | 95 | 6 | 59 | 6 | 166 | 13.04 | 1.88 | 60.8 |  |
| ***Thr, T*** | 39 | 1 | 37 | 6 | 83 | 6.52 | 1.16 | 54.2 |  |
| ***Trp, W*** | 8 | 0 | 6 | 0 | 14 | 1.1 | -1.64 | 57.1 |  |
| ***Tyr, T*** | 2 | 2 | 5 | 0 | 9 | 0.71 | -3.57 | 22.2 |  |
| ***Val, V*** | 25 | 0 | 31 | 1 | 57 | 4.48 | -1.40 | 45.6 |  |
| ***Overall*** | **736** | **52** | **438** | **47** | **1273** | **100** | **-** | **61.5** | |

The integrase insertion bias in close proximity of each tRNA was calculated as *Obs/Exp* where *Obs* is the proportion of specific *InTr* shapes (over the 1273 InTr shapes) and *Exp*, the proportion of the same tRNA out of the overall tRNA in 561 genomes. If the ratio *Obs/Exp* is < 1, the biais becomes *-Exp/Obs*. Note that Pseudo, Sup and Undef tRNAs (291 tRNAs from a total of 34596) were removed from our data. %AR, is the proportion of predicted autoregulated *InTr* shapes. Note that in four cases, the InTr shapes were found within the plasmids (eg. 2 in *Silici bacter_TM140* (NC008043, Ser-OC and Phe-TI), 1 in *Ralstonia eutropha* JM134 (NC_007336, Met-TI) and 1 in *Burkholderia phymatum STM 185* (NC_010625, Leu-OC).

**Table S3.** *InTr* codon shape frequencies and biases.

| ***tRNA*** | ***Codon*** | **% obs** | **% 561** | **Biais** | ***tRNA*** | ***Codon*** | **% Obs** | **% 516** | **Biais** |
| --- | --- | --- | --- | --- | --- | --- | --- | --- | --- |
| ***Ala, A*** | **GCT**  **GCC**  **GCA**  **GCG** | -  1.18  0.24  1,02 | -  1.96  4.56  0.72 | -  -1.66  -19.0  1.41 | ***Lys, K*** | **AAA**  **AAG** | 1.96  1.41 | 3.74  1.19 | -1.91  1.18 |
| ***Arg, R*** | **CGT**  **CGC**  **CGA**  **CGG**  **AGA**  **AGG** | 1.41  -  0.47  1.81  4.01  5.18 | 3.18  0.14  0.39  1.50  2.01  1.45 | -2.26  -  1.21  1.21  1.99  3.58 | ***Met, M*** | **ATG** | 8.41 | 7.88 | 1.07 |
| ***Asn, N*** | **AAT**  **AAC** | -  4.16 | -  3.49 | -  1.19 | ***Phe, F*** | **TTT**  **TTC** | -  6.68 | <10^-4^  2.43 | -  2.75 |
| ***Asp, D*** | **GAT**  **GAC** | -  0.47 | -  3.69 | -  -7.84 | ***Pro, P*** | **CCT**  **CCC**  **CCA**  **CCG** | -  2.59  1.18  0.86 | <10^-4^  1.33  2.22  1.14 | -  1.95  -1.88  -1.33 |
| ***Cys, C*** | **TGT**  **TGC** | -  1.96 | -  1.91 | -  1.03 | ***SelC*** | **TGA** | 2.91 | 0.49 | 5.88 |
| ***Gln, Q*** | **CAA**  **CAG** | 0.63  0.63 | 2.54  1.09 | -4.03  -1.73 | ***Ser, S*** | **TCT**  **TCC**  **TCA**  **TCG**  **AGT**  **AGC** | -  4.01  3.22  4.56  -  1.26 | <10^-4^  1.85  2.10  1.21  -  1.77 | -  2.16  1.54  3.76  -  -1.41 |
| ***Glu, E*** | **GAA**  **GAG** | 0.94  0.79 | 4  0.68 | -4.25  1.16 | ***Thr, T*** | **ACT**  **ACC**  **ACA**  **ACG** | -  1.18  1.41  3.93 | 0.04  1.96  2.35  1.28 | -  -1.66  -1.67  3.06 |
| ***Gly, G*** | **GGT**  **GGC**  **GGA**  **GGG** | -  2.04  0.63  4.79 | -  3.97  2.09  1.2 | -  -1.95  -3.31  4.0 | ***Trp, W*** | **TGG** | 1.10 | 1.82 | -1.66 |
| ***His, H*** | **CAT**  **CAC** | -  0.94 | -  1.87 | -  -1.99 | ***Tyr, Y*** | **TAT**  **TAC** | -  0.71 | -  2.53 | -  -3.57 |
| ***Ile, I*** | **ATT**  **ATC**  **ATA** | -  0.24  0.08 | <10^-4^  4.21  0.05 | -  -17.56  1.74 | ***Val, V*** | **GTT**  **GTC**  **GTA**  **GTG** | -  2.04  1.26  1.18 | <10^-4^  1.96  3.52  0.79 | -  1.04  -2.79  1.49 |
| ***Leu, L*** | **TTA**  **TTG**  **CTT**  **CTC**  **CTA**  **CTG** | 2.91  7.62  0.08  1.89  1.26  0.79 | 1.89  1.59  0.12  1.67  2.05  2.35 | 1.54  4.80  -1.55  1.13  -1.63  -2.97 |  |  |  |  |  |

For each tRNA, the *InTr* tRNA codon biais was computed as Obs/All where Obs, is the proportion of *InTr* tRNA codon shapes over the total number of *InTr* shapes and All is the proportion of the same Intr shape codon over the total number of tRNA codons in the 561 organisms. Threshold ratios for positive and negative biaises are [1] and [-1], respectively. One hundred and six uncertain codons, one TAA codon and two TAG codons (from Sup tRNA) were removed from the data (34887 codons from the 561 genomes with *InTr* shapes). <10^-4^, less than 0.0001. Negative and positive biases are marked by (-) and (+), respectively.

**Figure legends:**

**Figure S1**. Expression of the *att*L-*gfp* transcriptional fusion in the bacterial population.

LCB6007 (∆*intS*) / pJF119EH (0), LCB6007 / pJFi (+TorI) or ENZ1734 (*wt*) / pJF119EH (+IntS) strains were transformed with pUA66-gfp (empty vector, A.) and p*att*L-*gfp* (wt, B.). After an overnight aerobic growth in the presence of 1 mM of IPTG for *torI* induction, the average fluorescence of the bacteria was calculated (see Materials and Methods). Population distributions according to the average fluorescence are plotted.

**Figure S2:** *InTr* insertion biaises with respect to the tRNA codon.

For each tRNA, the *InTr* tRNA codon bias was computed as Obs/All where Obs, is the proportion of *InTr* tRNA codon shapes over the total number of *InTr* shapes and All is the proportion od the same Intr shape codon over the total number of tRNA codons in the 561 organisms. Threshold ratios for positive and negative biases are set to [1] and [-1], respectively. For more details, see Table S1.

Reference List

Boyd,E.F., Almagro-Moreno,S., and Parent,M.A. (2009). Genomic islands are dynamic, ancient integrative elements in bacterial evolution. Trends Microbiol. *17*, 47-53.

Fouts,D.E. (2006). Phage_Finder: automated identification and classification of prophage regions in complete bacterial genome sequences. Nucleic Acids Res. *34*, 5839-5851.

Williams,K.P. (2002). Integration sites for genetic elements in prokaryotic tRNA and tmRNA genes: sublocation preference of integrase subfamilies. Nucleic Acids Res. *30*, 866-875.


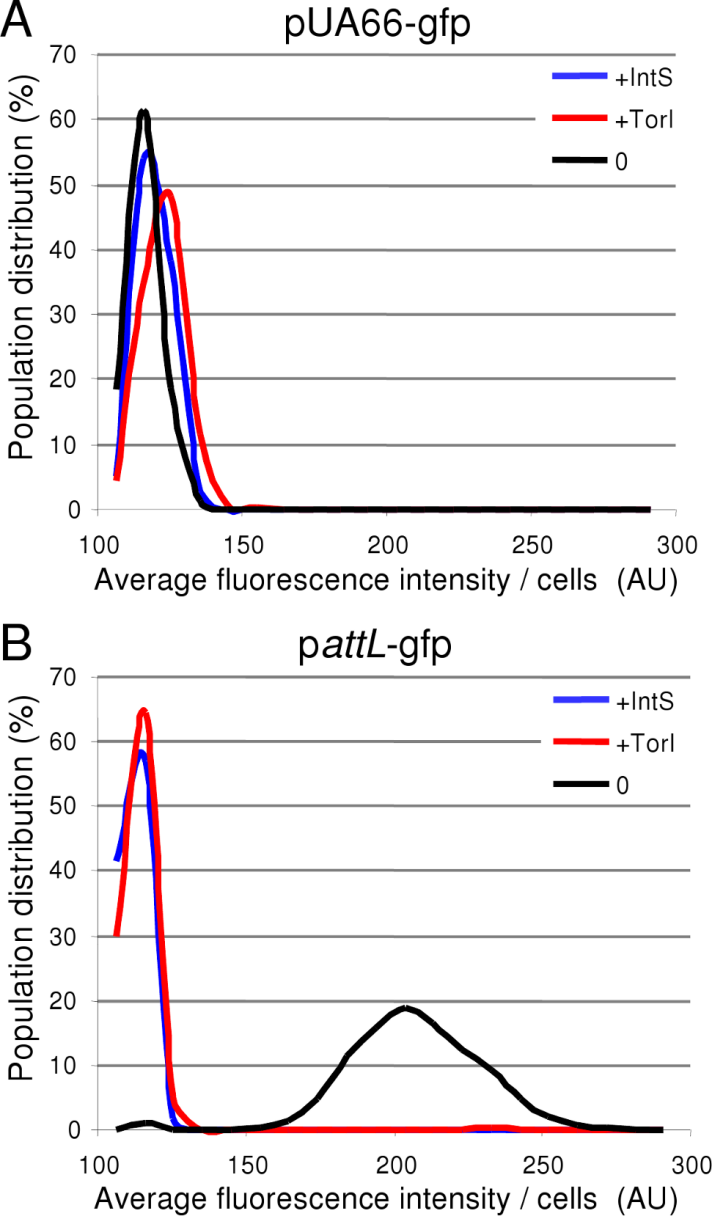


Figure S1


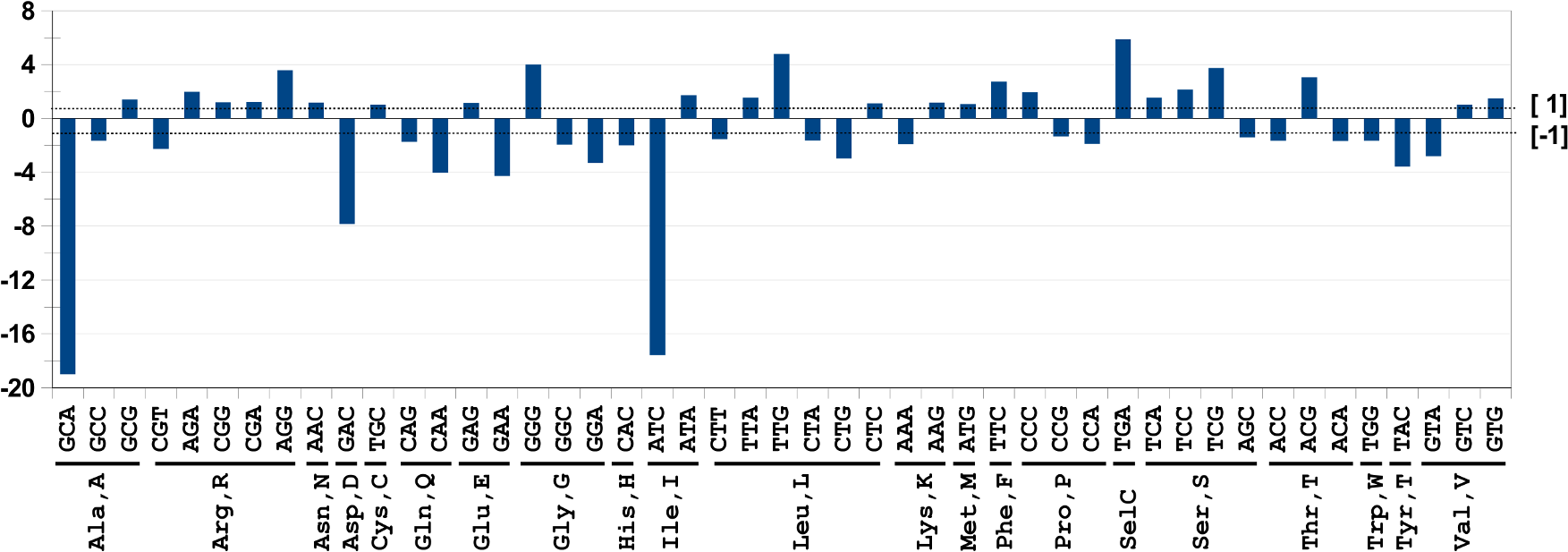


Figure S2
